# India-Asia collision as a driver of atmospheric CO_2_ in the Cenozoic

**DOI:** 10.1038/s41467-021-23772-y

**Published:** 2021-06-23

**Authors:** Zhengfu Guo, Marjorie Wilson, Donald B. Dingwell, Jiaqi Liu

**Affiliations:** 1 Key Laboratory of Cenozoic Geology and Environment, Institute of Geology and Geophysics, Chinese Academy of Sciences (CAS), Beijing, China; 2 CAS Center for Excellence in Life and Paleoenvironment, Beijing, China; 3 School of Earth and Environment, University of Leeds, Leeds, UK; 4 Department of Earth and Environmental Sciences, Ludwig-Maximilians-Universität, Munich, Germany

**Keywords:** Carbon cycle, Palaeoclimate, Geology, Tectonics

## Abstract

Deep Earth degassing is a critical forcing factor for atmospheric CO_2_ variations and palaeoclimate changes in Earth’s history. For the Cenozoic, the key driving mechanism of atmospheric CO_2_ variations remains controversial. Here we analyse three stages of collision-related magmatism in Tibet, which correspond temporally with the three major stages of atmospheric CO_2_ variations in the Cenozoic and explore the possibility of a causal link between these phenomena. To this end we present geochemical data for the three stages of magmatic rocks in Tibet, which we use to inform a model calculating the continental collision-induced CO_2_ emission flux associated with the evolving Neo-Tethyan to continental subduction over the Cenozoic. The correlation between our modelled CO_2_ emission rates and the global atmospheric CO_2_ curve is consistent with the hypothesis that the India-Asia collision was the primary driver of changes in atmospheric CO_2_ over the Cenozoic.

## Abstract (web summary)

“Earth degassing is a critical carbon source, but its contribution to Cenozoic atmospheric CO_2_ variations is not well known. Here, the authors analyse CO_2_ fluxes on the Tibetan Plateau and suggest that the India-Asia collision was the primary driver of changes in atmospheric CO_2_ over the past 65 Ma.”

## Introduction

Cooling of Earth’s climate during the Cenozoic is broadly thought to have been related to decreasing atmospheric CO_2_ concentrations^1–3^. The key factors leading to such decreases remain the subject of considerable debate^4–8^. Two hypotheses have been proposed. The first invokes an increasing rate of CO_2_ consumption by silicate weathering during the Cenozoic, caused by the uplift of the Tibetan Plateau^9–12^. The second focuses on a decreasing rate of CO_2_ release from Earth’s interior in the Cenozoic, attributed to the shutdown of the Neo-Tethyan decarbonation subduction factory during the India-Asia continent collision^7,8,13^. Despite the clear distinction between these two hypotheses, both contain the premise that the India-Asia collision is the ultimate cause of atmospheric CO_2_ concentration variations in the Cenozoic. Thus, a better understanding of the processes of India-Asia collision might well provide important constraints on controls of Cenozoic atmospheric CO_2_ variations.

Several studies^14–18^ indicate that Cenozoic magmatism and metamorphism in the Tibetan Plateau are reliable recorders of the evolution of India-Asia collision processes and the formation of the Himalayan-Tibetan orogen. Magmatic and metamorphic degassing, which are fundamentally linked to such plate tectonic processes, are important parts of the Earth’s deep carbon cycle on million-year time scales^8,19–21^. Thus, a better understanding of magmatic and metamorphic emissions in Tibet should bring with it the potential for providing critical constraints on explanations of atmospheric CO_2_ concentration variations in the Cenozoic. It is even possible that deep carbon cycle processes associated with the India-Asia collision might have served as critical drivers of both atmospheric CO_2_ concentration variations and palaeoclimatic changes in the Cenozoic^8,20^.

Along these lines of logic, recent studies have indicated that the convergence rate between the Indian and Asian continents, together with the magnitude and recycling efficiency of subducted Neo-Tethyan lithosphere, has exerted important controls on the amount of magmatic CO_2_ emissions and thereby global climate changes in the Cenozoic^19,22^. Further, enhanced magmatic CO_2_ degassing in the early Cenozoic due to subduction of a carbonate-rich component beneath the Tethyan-Himalayan orogen has been proposed as a cause of the late Paleocene-early Eocene climate warming^7,13,23^. Based on geological studies of the geodynamic evolution of the India-Asia subduction and collisional systems, Sternai et al. have modelled the climatic effects of Neo-Tethyan arc extinction^8^. They propose that waning volcanic degassing along the southern Eurasian margin might have resulted in the long-term palaeoclimate cooling in the Cenozoic. Finally, Anagnostou et al. have re-evaluated the possibility of a genetic relationship between global volcanism (including Neo-Tethyan arc volcanic activity), silicate weathering and atmospheric CO_2_ levels in the Eocene using their recently developed continuous CO_2_ record, concluding that enhanced volcanism is a potential driving factor of high levels of atmospheric CO_2_ during the EECO (early Eocene climatic optimum; 49–53 Ma)^24^.

CO_2_ outgassing due to metamorphic decarbonation along the Himalayan orogenic belt has also been inferred to have contributed substantially to atmospheric CO_2_ levels in the Cenozoic^25–27^. Yet the importance of metamorphic CO_2_ emissions in controlling the evolution of atmospheric CO_2_ levels over the last 65 Ma, and hence their role in the evolution of Cenozoic climate, remains poorly constrained^28–30^. Despite the fact that the above studies have indicated significant contributions from Tibetan-Himalayan magmatic and metamorphic outgassing to global climate changes, there remains a lack of continuous and quantitative calculations of both magmatic and metamorphic outgassing fluxes from early to late over the whole Cenozoic. This has, in turn, precluded a more critical analysis of the tectonic processes involved in the uplift of the Tibetan Plateau as a source or sink of atmospheric CO_2_^31,32^.

Here, in order to quantify the further exploration of relationships between the tectonic evolution of Tibet and global atmospheric CO_2_ variations, we develop a model capable of generating calculated fluxes from Tibetan magmatic and metamorphic CO_2_ outgassing over the last 65 Ma. The model is constrained by new and previously published geochemical and geochronological data for magmatic rocks in the Plateau. Based on the results of our model, together with a determination of the geodynamic evolution of the continental collision in the formation of the Himalayan-Tibetan orogen, we propose that the magmatic–metamorphic CO_2_ degassing generated by the closure of the Neo-Tethyan Ocean and the India-Asia collision drove global Cenozoic CO_2_ variations and climate changes. The results presented here comprise a comprehensive set of evidence from independent observational data sets on the tectonic evolution of Tibet which bear on the question of the controlling factors of atmospheric CO_2_ concentration variations in the Cenozoic.

## Results

### Three-stage evolution of Cenozoic magmatism in Tibet

**Fig. 1:**
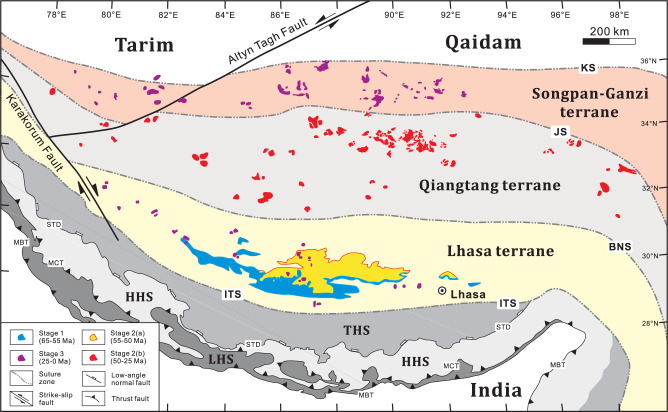
Spatio-temporal distribution of Cenozoic three-stage magmatism in the Tibetan Plateau. Spatial distribution, geochronological and geochemical data for the magmatic rocks are taken from Supplementary Data 1 and 2. Stage 1 (65–55 Ma) formed in south Tibet (blue fields); Stage 2 (55–25 Ma) occurred in south-central Tibet, which includes Stage 2(a) in south Tibet (55–50 Ma; yellow fields) and Stage 2(b) in central Tibet (50–25 Ma; red fields); Stage 3 is distributed in south and north Tibet (purple fields). BNS Bangong–Nujiang suture, HHS higher Himalayan sequence, ITS Indus-Tsangpo suture, JS Jinsha suture, KS Kunlun suture, LHS lesser Himalayan sequence, MBT main boundary thrust, MCT main central thrust, STD south Tibetan detachment, THS Tethyan Himalayan sequence.

**Fig. 2:**
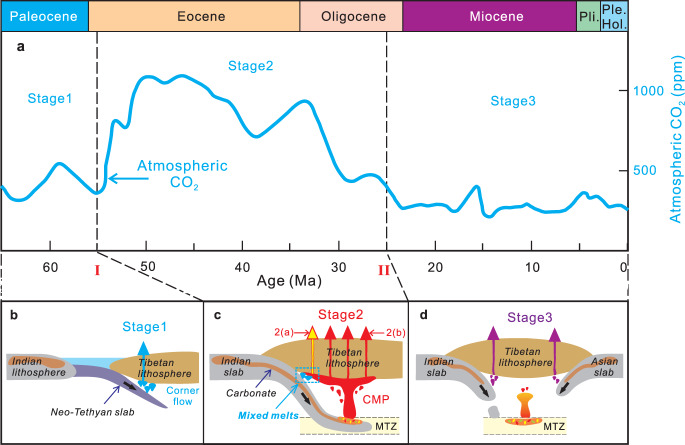
Trilogy of plate subduction related to three-stage evolution of petrogenesis of collision-related Tibetan magmatism and global atmospheric CO_2_ concentration variations in the Cenozoic. **a** Three-stage variations of atmospheric CO_2_ concentrations in the Cenozoic. The data are from ref. ^2^. **b**–**d** Three-stage evolution of the petrogenesis of collision-related Tibetan magmatism in the Cenozoic (see details in Supplementary Figs. 1–6). Neo-Tethyan oceanic lithosphere subduction in (**b**) resulted in corner flow and intermediate atmospheric CO_2_ levels of Stage 1 (from 65 to 55 Ma) in (**a**). Indian continental lithosphere subduction in (**c**) resulted in a large-scale CMP upwelling and high atmospheric CO_2_ concentrations of Stage 2 (from 55 to 25 Ma) in (**a**). India and Asia subduction in (**d**) resulted in a small-scale of opposing mantle convection and low atmospheric CO_2_ levels of Stage 3 (from 25 to 0 Ma) in (**a**). The mixed melts in (**c**) denote a plume-wedge interaction in Stage 2(a) from 55 to 50 Ma. The black dashed lines labelled I and II denote the first-step and second-step transformation of the mantle source region of the Tibetan magmas during the India-Asia collision (Supplementary Figs. 1–6), respectively. I: the first-step transformation from Neo-Tethyan oceanic lithospheric subduction-induced silicate-rich metasomatism to India subduction-induced carbonate-rich metasomatism, II: the second-step transformation from India subduction-induced carbonate-rich metasomatism to dual India and Asia subduction-induced silicate-rich and carbonate-rich metasomatism.

Cenozoic magmatism has occurred across the entire Tibetan Plateau^14–16,33^. Our new geochemical data for 56 samples of primitive magmatic rocks, together with previously published geochronological and geochemical data for 231 samples, cover all Cenozoic volcanic fields (i.e., 55 volcanic fields shown in Fig. 1) in the Tibetan Plateau (Supplementary Data 1 and Data 2). Based on these data, Cenozoic magmatic activity can be subdivided into three stages—from early to late—on the basis of compositional differences (Fig. 1) whose origins have been linked to the mantle source regions of the magmas (Fig. 2). The Stage 1 magmas (65–55 Ma) are Andean-type igneous rocks in southern Tibet (Fig. 1), which are interpreted to result from Neo-Tethyan lithospheric subduction (Fig. 2b, Supplementary Figs. 1 and 2). Their Sr–Nd–Pb isotopic compositions indicate that their mantle source regions are dominated by enriched components derived from subducted silicate-rich terrigenous sediments (Supplementary Fig. 3). The Stage 2 magmatic rocks (55–25 Ma) are composed of large-scale lava flows and pyroclastic deposits in central-southern Tibet (Fig. 1)^14,33^. They have been interpreted to result from upwelling of a mantle transition zone (MTZ)-derived carbonated asthenospheric mantle plume (CMP), induced by the northward subduction of the Indian slab (Fig. 2c, Supplementary Figs. 1–5). Our Sr–Nd–Pb isotopic data indicate that the mantle source of the parental magmas was characterised by extensive enrichment in a subducted Indian carbonate-rich component (Supplementary Figs. 3 and 6), with a much higher carbonate component than the sources of Stage 1 and 3 magmas. The Stage 3 magmas (25 Ma-present) is characterised by small-volume K-rich lava flow series^15^, which are sporadically distributed in the south and north of the Plateau (Fig. 1). They are interpreted to result from dual polarity subduction of the Indian and Asian continents (Fig. 2d, Supplementary Figs. 4 and 5). The Sr–Nd–Pb isotopic compositions of Stage 3 magmatic rocks indicate that they result from subduction of silicate-rich continental material (Supplementary Fig. 6)^16^.

### Temporal correlation of Tibetan magmatism with atmospheric CO_2_

The transition from ocean-continent subduction in Stage 1 (i.e., pre-collision) through continent-continent subduction in Stage 2 (i.e., syn-collision) to double continent underthrusting in Stage 3 (i.e., late-collision) clearly influences the nature and compositions of the subducting materials and thus the magmas produced and eventually erupted. Comparing the evolution of magmatism in the Tibetan Plateau (Fig. 1) versus the atmospheric CO_2_ curve throughout the Cenozoic, it can be seen that global atmospheric CO_2_ concentration variations^2^ may be subdivided into three stages (Fig. 2a). Stage 1 exhibits flat and intermediate CO_2_ contents from 65 to 55 Ma (400–450 ppm average), Stage 2 displays elevated CO_2_ concentrations from 55 to 25 Ma (900  ppm average) and Stage 3 demonstrates low CO_2_ atmospheric concentrations with small-scale variations during the period 25 Ma to the present (300 ppm average).

### Calculations of continental collision-derived CO_2_ flux

The correlation throughout the Cenozoic of the evolution of magmatism in Tibet and atmospheric CO_2_ begs the question of whether there is a mechanistic link between the two phenomena (Fig. 2). To evaluate quantitatively the plausibility of a link between these observations, we have developed a continental collision-derived CO_2_ flux model (CCFM). With this model, we have estimated the total flux of CO_2_ emissions from magmatism and metamorphism during the closure of the Neo-Tethyan Ocean and the India-Asia collision across the whole of the Cenozoic. For a detailed description of our calculation procedures see “Methods”. Results of the CCFM include the following two salient features, firstly, the achievement of time-series of model calculations and secondly, the estimation of fluxes of collision-derived CO_2_ emission throughout the Cenozoic.

**Fig. 3:**
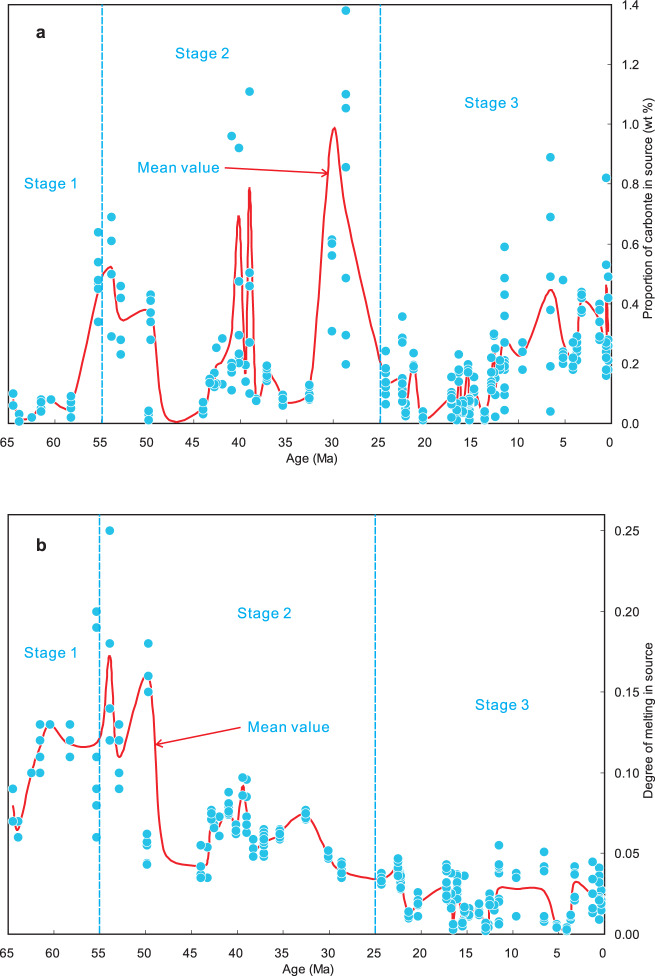
Three-stage evolution of carbonate contents and melting degrees. Each filled blue dot denotes a calculation result of the carbonate content in (**a**) and melting degree in (**b**) by CCFM based on a set of geochemical data (including major element, trace element and Sr–Nd–Pb isotope composition) of each sample from 287 samples (for detailed calculation procedures see Supplementary Data 2 and “Methods”), which have been distributed in 55 volcanic fields in Tibetan Plateau in the Cenozoic (Fig. 1). The mean value of the modelling results of these geochemical parameters in (**a**) and (**b**) in each volcanic field is shown as a solid red curve (for detailed calculation results of the mean values see Table 1).

**Fig. 4:**
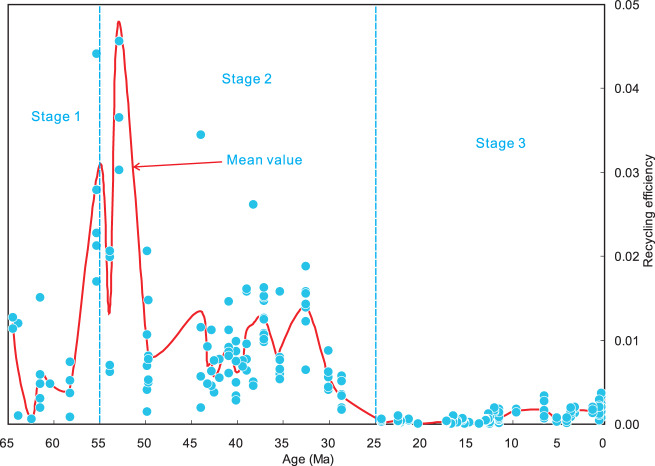
Three-stage evolution of recycling efficiency. Each filled blue dot denotes a calculation result of the recycling efficiency by CCFM based on a set of geochemical data (including major element, trace element and Sr–Nd–Pb isotope composition) of each sample from 287 samples (for detailed calculation procedures see Supplementary Data 2 and “Methods”), which have been distributed in 55 volcanic fields in Tibetan Plateau in the Cenozoic (Fig. 1). The mean value of the recycling efficiencies in each volcanic field is shown as a solid red curve (for detailed calculation results of the mean values see Table 1).

We have selected the mean values of the eruptive ages in all volcanic fields of the Tibetan Plateau to act as a time-series for expressing our modelled calculation results from early to late Cenozoic. Statistical calculations using geochronological data for the magmatic rocks (Supplementary Data 1) yield the mean value (Table 1) of the eruptive ages in each volcanic field of the Tibetan Plateau from ~65 Ma to the present. These reveal a near-continuous time-series of mean values of the ages in all volcanic fields in the Cenozoic (Figs. 3 and 4). The mean ages (Table 1 and Fig. 3) in the different volcanic fields from early to late Cenozoic are used as our modelling time-points in order to express subsequently a time series of the CCFM results.

**Table 1:** Mean value of eruption age, geochemical parameter and CO_2_ flux from each Cenozoic volcanic field in Tibet.

No. of the volcanic field	The average age in each volcanic field (Ma)	Average carbonate in the source (%)	Average silicate in the source (%)	The average degree of melting in source	Average proportion of H^+^ in source (H^+^_i_)	The average value of recycling efficiency	The average value of magmatic CO_2_ flux (Pg/year)	The average value of metamorphic CO_2_ flux (Pg/year)	The average value of total flux (Pg/year)
1	0.33	0.3067	3.5350	0.0205	0.0002039	0.0023	0.0206		0.0206
2	0.56	0.4625	5.5625	0.0240	0.0002082	0.0019	0.0173		0.0173
3	0.59	0.2075	3.5175	0.0278	0.0002278	0.0016	0.0138		0.0138
4	1.30	0.3450	5.5417	0.0245	0.0001977	0.0016	0.0142		0.0142
5	3.24	0.4080	7.4000	0.0322	0.0002011	0.0017	0.0147		0.0147
6	3.72	0.2567	1.7350	0.0108	0.0001827	0.0015	0.0130		0.0130
7	4.16	0.2100	1.7800	0.0032	0.0001806	0.0004	0.0078		0.0078
8	5.20	0.2740	2.6900	0.0050	0.0001986	0.0005	0.0092		0.0092
9	6.62	0.4467	5.1600	0.0268	0.0002103	0.0017	0.0327		0.0327
10	9.61	0.2300	4.4433	0.0280	0.0002033	0.0016	0.0348		0.0348
11	11.50	0.2821	10.3564	0.0285	0.0001638	0.0008	0.0152		0.0152
12	12.03	0.1933	1.6500	0.0163	0.0001900	0.0017	0.0342		0.0342
13	12.53	0.1298	6.8450	0.0220	0.0001628	0.0004	0.0082		0.0082
14	12.68	0.2543	1.4900	0.0050	0.0002040	0.0008	0.0162		0.0162
15	12.95	0.1483	1.0433	0.0040	0.0001782	0.0005	0.0100		0.0100
16	13.70	0.0272	4.5778	0.0143	0.0001642	0.0001	0.0031		0.0031
17	14.80	0.1120	5.3825	0.0130	0.0001714	0.0003	0.0094		0.0094
18	15.29	0.0588	5.3777	0.0162	0.0001710	0.0002	0.0059		0.0059
19	15.47	0.1823	1.9633	0.0067	0.0001963	0.0006	0.0219		0.0219
20	16.04	0.0480	5.8371	0.0300	0.0001822	0.0002	0.0088		0.0088
21	16.47	0.1800	1.4075	0.0045	0.0001861	0.0006	0.0207		0.0207
22	16.70	0.0384	6.6300	0.0234	0.0001783	0.0001	0.0042		0.0042
23	17.23	0.0855	7.7708	0.0299	0.0001749	0.0003	0.0113		0.0113
24	20.35	0.0187	3.7850	0.0190	0.0001657	0.0001	0.0031		0.0031
25	21.34	0.1990	4.2880	0.0118	0.0001690	0.0005	0.0188		0.0188
26	22.22	0.0477	5.6090	0.0340	0.0001755	0.0003	0.0133		0.0133
27	22.53	0.1596	13.1982	0.0423	0.0001737	0.0005	0.0218		0.0218
28	24.35	0.1449	11.6257	0.0340	0.0001688	0.0004	0.0191		0.0191
29	28.67	0.7088	7.4388	0.0390	0.0001868	0.0034	0.0838		0.0838
30	30.13	0.9656	6.2200	0.0490	0.0001876	0.0058	0.1450		0.1450
31	32.57	0.1091	0.5136	0.0741	0.0002707	0.0139	0.3446		0.3446
32	35.41	0.0698	0.5325	0.0628	0.0002685	0.0088	0.2678		0.2678
33	35.43	0.0890	0.6800	0.0610	0.0002760	0.0071	0.2169		0.2169
34	37.14	0.1741	0.6135	0.0572	0.0001863	0.0128	0.4129		0.4129
35	38.30	0.0780	0.5533	0.0533	0.0002812	0.0119	0.2750		0.2750
36	39.00	0.4888	3.3620	0.0770	0.0001981	0.0111	0.2566		0.2566
37	39.43	0.1675	1.9150	0.0915	0.0002045	0.0073	0.1674		0.1674
38	40.18	0.3915	4.3383	0.0652	0.0001881	0.0062	0.1435		0.1435
39	40.95	0.3008	1.8067	0.0788	0.0002072	0.0097	0.2227		0.2227
40	41.95	0.2075	2.1800	0.0670	0.0002019	0.0066	0.1529		0.1529
41	42.59	0.1930	2.0950	0.0670	0.0002095	0.0057	0.1314		0.1314
42	42.84	0.1403	1.4033	0.0743	0.0002058	0.0074	0.1704		0.1704
43	43.32	0.1425	0.7950	0.0445	0.0002682	0.0070	0.2181		0.2181
44	44.00	0.0500	0.3975	0.0423	0.0002851	0.0134	0.5363		0.5363
45	49.87	0.0258	0.2096	0.0522	0.0002641	0.0088	0.3506		0.3506
46	49.70	0.3660	7.9180	0.1600	0.0000454	0.0082	0.3706	3.0931	3.4637
47	52.90	0.3475	0.5125	0.1100	0.0000649	0.0479	2.2950	4.4256	6.7206
48	53.90	0.5225	9.4850	0.1725	0.0000475	0.0135	0.6448	3.2403	3.8851
49	55.37	0.4867	2.1383	0.1217	0.0000253	0.0309	0.3244	1.7259	2.0503
50	58.30	0.0575	1.9300	0.1175	0.0000011	0.0043	0.0012		0.0012
51	60.43	0.0800	2.0600	0.1300	0.0000011	0.0049	0.0014		0.0014
52	61.50	0.0620	1.6540	0.1160	0.0000010	0.0062	0.0018		0.0018
53	62.50	0.0200	3.1800	0.1000	0.0000014	0.0006	0.0002		0.0002
54	63.89	0.0180	0.2650	0.0650	0.0000008	0.0065	0.0019		0.0019
55	64.47	0.0800	0.4800	0.0800	0.0000010	0.0121	0.0035		0.0035

The mean value of eruption age, geochemical parameter and CO_2_ flux from each Cenozoic volcanic field in Tibet shown above are achieved and calculated using original data in Supplementary Data 1 and Data 2.

**Fig. 5:**
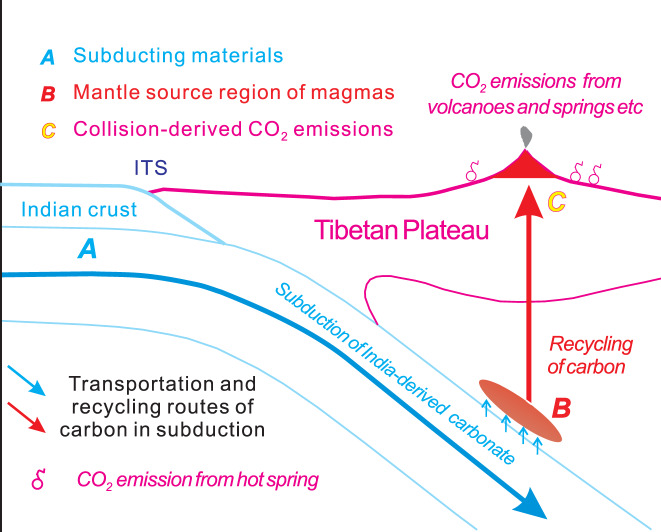
A cartoon of transportation and recycling routes of carbon-rich components in Neo-Tethyan Ocean and Indian continent subduction in the Cenozoic. A: Subducting materials. B: Mantle source region of the Cenozoic magmas in Tibet. C: Collision-derived CO_2_ emissions (including metamorphic and magmatic CO_2_ emissions). ITS Indus-Tsangpo suture.

We propose that CO_2_ emissions (Fig. 5) from the different volcanic fields of the Tibetan Plateau in the Cenozoic may act as fluxes of collision-derived CO_2_ production from early to late in our model calculations. In order to estimate the flux of collision-derived CO_2_ emission in each volcanic field from early to late in the Cenozoic, we first carried out a determination of the compositions of the end-members in the mantle source region of the magmas in the volcanic fields of the Tibetan Plateau (Fig. 1), using new and previously published geochemical data for the magmatic rocks in the corresponding volcanic field from 65 Ma to the present (Supplementary Data 2). Our detailed results (Supplementary Figs. 3 and 6; Table 2) indicate that the compositions of the source region of the magmas in the different volcanic fields throughout the Cenozoic may be subdivided into three stages from early to late. Stage 1 (65–55 Ma) is largely composed of depleted mid-oceanic ridge basalt (MORB)-source mantle (DMM), silicate-rich global subducting sediment (GLOSS)^34^ and India-derived carbonate sediments. Stage 2 (55–25 Ma) is dominated by DMM, India-derived carbonate and silicate components. Stage 3 (from 25 Ma to the present) is mainly composed of DMM, as well as Asia- and India-derived silicate sediments. Further, the dominant enrichment agents derived from the subducting materials (Table 2; Fig. 5) may also be subdivided into these three stages, which evolved from the NeoTethys-derived GLOSS-dominated components in Stage 1 through Indian subduction-induced carbonate-rich melts in Stage 2 to India- and Asia-derived silicate-rich components in Stage 3.

**Table 2:** Compositions of the end-members in the source regions of the magmas.

End-member	Sr (ppm)	Nd (ppm)	Pb (ppm)	(^87^Sr/^86^Sr)_i_	(^143^Nd/^144^Nd)_i_	(^206^Pb/^204^Pb)_i_
DMM	33.0	1.925	0.16	0.7040	0.51275	18.150
GLOSS	327	27.0	19.9	0.7173	0.51218	18.913
India-derived silicate	122	29.7	16.5	0.7490	0.51145	19.20
India-derived carbonate	1927	741	125	0.7095	0.5127	19.10
Asia-derived silicate	683	46.0	29.0	0.7109	0.5118	18.30

Secondly, we estimated proportions of the above end-members and degrees of partial melting in the mantle source region of the magmatic rocks in each volcanic field of the Tibetan Plateau in the Cenozoic from early to late (Table 1 and Fig. 3), using three-component mixing calculations of Sr–Nd–Pb isotope compositions^35^ and trace element modelling calculations^36^. Based on these geochemical model calculation results for the 55 volcanic fields throughout the Cenozoic in Tibet (Supplementary Data 2), both the proportions of the carbonate-rich component and the degrees of partial melting in the source region may be subdivided into three stages (Table 1, Figs. 3 and 4). Stage 1 (65–55 Ma) exhibits low proportions of the carbonate-rich component (0.018–0.080%), high degrees of melting (6.50–13.00%) and intermediate recycling efficiencies (0.06–1.21%). Stage 2 (55-25 Ma) exhibits high contents of the carbonate-rich component (0.026–0.966%), intermediate degrees of melting (3.90–17.25%) and high recycling efficiencies (0.03–4.79%). Stage 3 (25 Ma to the present) displays intermediate proportions of the carbonate-rich component (0.019–0.463%), low degrees of melting (0.32–4.23%) and low recycling efficiencies (0.01–0.23%).

**Fig. 6:**
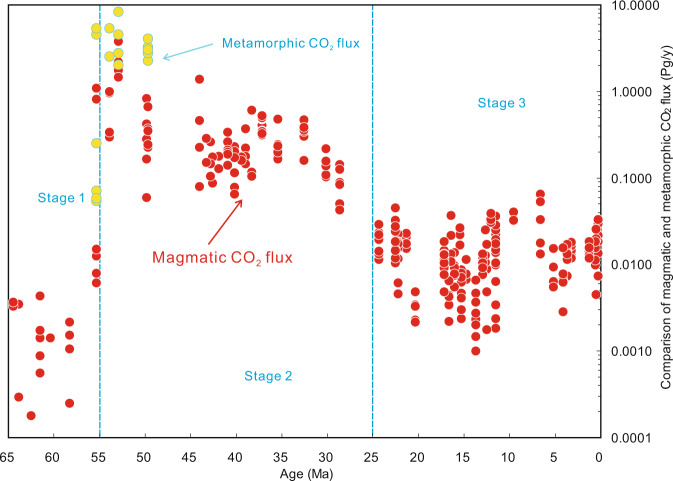
Three-stage evolution of magmatic and metamorphic CO_2_ outgassing rates. Each filled red and yellow dot denotes a calculation result of the magmatic and metamorphic CO_2_ outgassing rate by CCFM, respectively, based on a set of geochemical data (including major element, trace element and Sr–Nd–Pb isotope composition) of each sample from 287 samples (for detailed calculation procedures see “Methods” and Supplementary Data 2), which have been distributed in 55 volcanic fields in Tibetan Plateau in the Cenozoic (Fig. 1). 1 Pg = 10^15^ g.

**Fig. 7:**
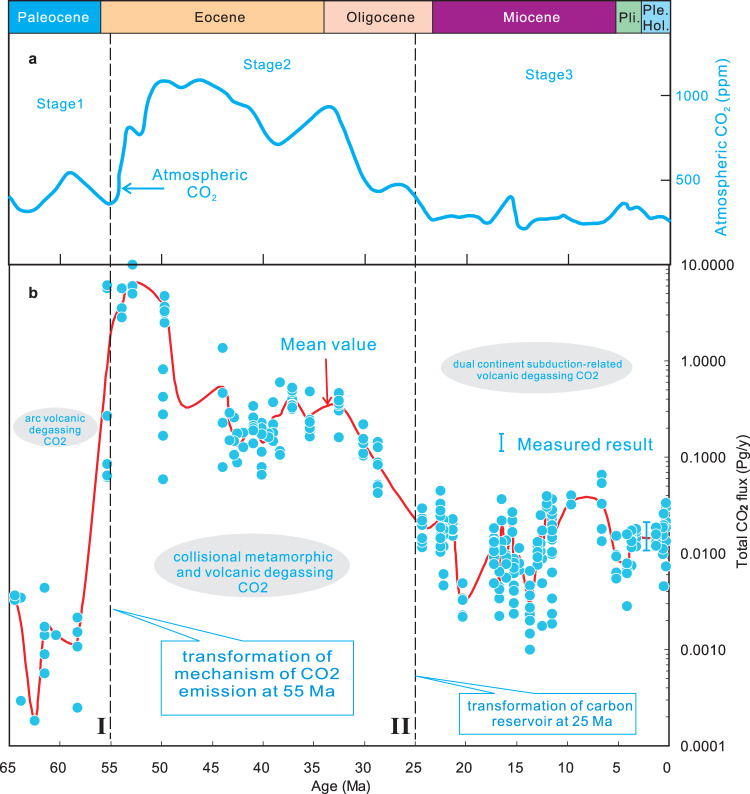
Three-stage evolution of the quantitative calculation results of CO_2_ flux derived from India-Asia collision-induced degassing using CCFM and global atmospheric CO_2_ level variations in the Cenozoic. **a** Three-stage variations of atmospheric CO_2_ concentrations in the Cenozoic. The data are from ref. ^2^. **b** Our CCFM modelling results, indicating a similar three-stage pattern of rates of CO_2_ released from the India-Asia collisional orogen in the Cenozoic. The measured data for the CO_2_ degassing flux in Tibet^45,51^, which is shown by a blue bold line, is consistent with our modelling calculation results. Each filled blue dot in (**b**) denotes a calculation result of India-Asia collision-related mantle degassing (including the magmatic and metamorphic CO_2_ outgassing) rate by CCFM based on a set of geochemical data (including major element, trace element and Sr–Nd–Pb isotope composition) of each sample from 287 samples (for detailed calculation procedures see “Methods” and Supplementary Data 2), which have been distributed in 55 volcanic fields in Tibetan Plateau in the Cenozoic (Fig. 1). The mean value of the collision-induced CO_2_ degassing rates in each volcanic field is shown as a solid red curve (for detailed calculation results of the mean values see Table 1). 1 Pg = 10^15^ g. The black dashed lines labelled I and II denote the first-step and second-step transformation of the deep carbon cycle processes during India-Asia collision, respectively. I: the first-step transformation from continental arc magmatism-induced CO_2_ degassing to collision-related metamorphic and magmatic CO_2_ emission; II: the second-step transformation from a carbonate platform-supplying carbon reservoir to a subducting silicate-rich continental material-supplying carbon reservoir.

Thirdly, we calculated the collision-derived rates of CO_2_ emissions from Cenozoic magmatism and metamorphism in the Tibetan Plateau, which indicate that the CO_2_ degassing fluxes range from ~0.0001 to 10 Pg (1Pg = 10^9^ Tons) in the various volcanic fields of Tibet over the last ~65 M year (Table 1 and Fig. 6). The model calculations yield a time series for collision-derived CO_2_ degassing rates from these volcanic fields in the Cenozoic (Fig. 7b). The CO_2_ degassing rates may also be divided into three stages (Table 1 and Fig. 7b). Stage 1 (65–55 Ma) exhibits low fluxes of degassing CO_2_ (0.0002–0.0035 Pg/year) with an overall increasing trend in the later period of this stage. Stage 2 (55–25 Ma) displays high rates of outgassing CO_2_ (0.0838–6.7206 Pg/year) with an increasing flux from 55 to 50 Ma in Stage 2(a) followed by a decreasing flux from 50 to 25 Ma in Stage 2(b). Sr–Nd–Pb isotope compositions (Supplementary Fig. 3) indicate that the flux of India-Asia collision-induced metamorphic CO_2_ emissions rose to one order of magnitude higher than that of magmatic degassing in Stage 2(a) (Table 1 and Fig. 6; Supplementary Data 2). Stage 3 (25 Ma to the present) reveals intermediate CO_2_ emissions (0.0031–0.0348 Pg/year) with small-scale variations. A peak value of the CO_2_ degassing rates (~7 Pg/year) occurred at ca. 52 Ma in the Cenozoic (Table 1 and Fig. 7b).

Our model calculation results indicate that a three-stage evolution of nature (Table 2) and compositions (Table 1 and Fig. 3) of the source regions of the magmatic rocks and the corresponding modelled CO_2_ emission curve (Fig. 7b) correlates very well with that of the global atmospheric CO_2_ curve from 65 Ma to the present (Fig. 7a). The high proportions of the carbonate-rich component (Table 1 and Fig. 3) and high fluxes of CO_2_ degassing (Table 1 and Fig. 7b) are consistent with high atmospheric CO_2_ concentrations in Stage 2 (Fig. 7a), whilst low-intermediate proportions of the carbonate-rich component (Table 1 and Fig. 3) and fluxes of degassing CO_2_ (Table 1 and Fig. 7b) coincide in time with low-intermediate atmospheric CO_2_ levels in Stages 1 and 3 (Fig. 7a).

## Discussion

The strong correlation between magmatic activity, source region composition, and modelled CO_2_ outgassing rate on the one hand and atmospheric CO_2_ concentration variations on the other is fully consistent with a causal link between India-Asia collision-induced tectonic evolution and global CO_2_ level variations–variations which very probably drove the long-term palaeoclimate changes inferred for the Cenozoic. The details of three-stage connections between tectonic evolution, atmospheric CO_2_ variations and palaeoclimate changes in the India-Asia collision are then as follows.

Stage 1 (65–55 Ma) was characterised by northward Neo-Tethyan Ocean lithospheric subduction (Fig. 2b), which resulted in partial melting of silicate-dominated metasomatised mantle and corner flow in the mantle wedge (Fig. 2b), forming the Stage 1 magmatic rocks in south Tibet (Fig. 1). Sr–Nd–Pb isotopic mixing model calculations (Table 1 and Fig. 3) indicate that the amount of subduction-derived carbonate-rich component in the mantle source region of the magmatic rocks is low (0.08%) between 65 and 55 Ma in Stage 1. This led to the occurrence of a continental arc volcanism-induced, low CO_2_ degassing flux (Table 1 and Fig. 7b), resulting in intermediate atmospheric CO_2_ levels from 65 to 55 Ma (Fig. 7a).

Stage 2 (55–25 Ma) was characterised by the occurrence of extensive pyroclastic and lava flow activity in Tibet (Fig. 1)^16,24,37^, which we take as important physical evidence of the potential for driving high levels of atmospheric CO_2_ during this period. Sr–Nd–Pb isotope studies indicate that there is a strong signature of the Tethyan carbonate platforms and carbonate-rich basins on the northern margin of the subducted Indian continent in the mantle source region (Fig. 2c; Supplementary Figs. 3 and 6). Three-component mixing model calculations of the Sr–Nd–Pb isotope compositions demonstrate that their source region exhibits a much higher proportion of the carbonate-rich component than those in Stages 1 and 3 (Table 1 and Fig. 3). We, therefore, infer a very high proportion of a carbonate-rich component in the subducting materials of Stage 2 (Fig. 5), whose composition is consistent with that of the Tethyan carbonate platforms and carbonate-rich basins on the northern margin of India. Stage 2 displays a high recycling efficiency of subducted carbonate-rich component (Table 1 and Fig. 4). The CCFM calculation results indicate a one to two orders of magnitude increase in CO_2_ degassing flux into the atmosphere in Stage 2 compared with Stages 1 and 3 (Table 1 and Fig. 7b). We note that our estimated CO_2_ flux (~7 Pg/year; Table 1 and Fig. 7b) in Stage 2 (from 55 to 25 Ma) is consistent with CO_2_ contributions from modern anthropogenic emissions^38^, and thus high enough to induce substantial changes in atmospheric CO_2_ concentration. We thus propose that the subduction of the carbonate-rich component in Stage 2 (Figs. 2 and 3) may have initiated and maintained the highest CO_2_ outgassing flux (Table 1 and Fig. 7b) and possibly resulting the highest atmospheric CO_2_ concentrations from 55 to 25 Ma in the entire Cenozoic (Fig. 7a).

Based on the geochemical composition of the magmatic rocks and mechanism of CO_2_ outgassing (Figs. 2 and 7b), Stage 2 (55–25 Ma) may further be subdivided into Stage 2(a) (55–50 Ma) and 2(b) (50–25 Ma) from early to late (Fig. 1). Stage 2(a) (55–50 Ma) comprised an initial period of Indian subduction, which resulted from an interaction (labelled mixed melts in Fig. 2c) between southward spreading in the head of upwelling of the CMP and GLOSS-rich mantle domains metasomatised by NeoTethys-derived components in the mantle wedge (Supplementary Fig. 3), that we call a plume-wedge interaction. This interaction has resulted in the large-scale magmatic CO_2_ emissions from 55 to 50 Ma in Stage 2(a) (Table 1) and an abruptly jump of ca. two orders of magnitude in magmatic outgassing from Stage 1 to Stage 2(a) (Fig. 6). Furthermore, the plume-wedge interaction may have reactivated a vast long hidden, deep carbon reservoir^20^ in the mantle wedge caused by the previous subduction of the Neo-Tethys Ocean lithosphere, leading to metamorphic CO_2_ emitted into the atmosphere from 55 to 50 Ma through the following carbonate dissolution reaction (1)^20,39^:

1 $${\rm{Ca}}{\rm{CO}}_{3}({\rm{wedge}})+{\rm{2H}}^{+}({\rm{plume}}) \to 	[{\rm{H}}_{2}{\rm{O}}+{{\rm{Ca}}}^{2+}]({\rm{fluid}}-{\rm{rich}}\,{\rm{wedge}})\\ 	+{\rm{CO}}_{2}({\rm{vapour}})$$

where CaCO_3_ (wedge) represents a deep carbon reservoir in the mantle wedge whose origin lies in previous long-term subduction of the Neo-Tethys Ocean lithosphere; H^+^ (plume) denotes an H^+^-rich component in the CMP, which results from the presence of water-containing residual minerals in the mantle source region of the magmatic rocks in Tibet (Table 1 and Supplementary Data 2); [H_2_O + Ca^2+^] (fluid-rich wedge) refers to H_2_O-rich characteristics in the composition of the Tibetan mantle wedge modified by the above metamorphism during a continental collision, which has further been corroborated by recent geophysical studies (e.g., ref. ^40^) and; CO_2_ (vapour) is the metamorphic carbon emission resulting from India-Asia collision in Stage 2(a) at 55–50 Ma.

It should be noted that the mechanism of CO_2_ production by metamorphic dissolution reaction (Eq. (1)), recently proposed for subduction zones^20,39^, is different from that of the traditional decarbonation reaction (Eq. (2))^25–27^.

2 $${{\rm{CaCO}}}_{3}+{{\rm{SiO}}}_{2}\to {{\rm{CaSiO}}}_{3}+{{\rm{CO}}}_{2}$$

which has been the focus of previous studies^25–27^ documenting Tibetan metamorphic CO_2_ emissions. The present metamorphic dissolution reaction (Eq. (1)) can consume the deep carbon reservoir previously preserved in the mantle wedge beneath Tibet and contribute up to 4.4 Pg/year of CO_2_ to the atmosphere during the period 55–50 Ma in Stage 2(a) (Table 1). The flux of metamorphic dissolution-induced CO_2_ outgassing (Eq. (1)), which may peak at ca. one order of magnitude higher than that of magmatic degassing (Table 1 and Fig. 6; Supplementary Data 2), displays an increase from 55 to 50 Ma (Fig. 7b). This is consistent with the notion that this continental collision-related metamorphic dissolution-induced outgassing resulted in increases in the atmospheric CO_2_ levels and resulting climate warming from 55 to 50 Ma in Stage 2(a)^1,2,8,23^. Thus, this metamorphic contribution (Eq. (1)) may dominate metamorphic outgassing in Tibet from 55 to 50 Ma and thereby have played a significant role in controlling global atmospheric CO_2_ concentration variations and Earth’s climate in Stage 2(a). A similar conclusion has been reached from the study of subducted rocks on the Greek islands of Syros and Tinos, where the carbonate dissolution reaction (Eq. (1)) has been proposed to have released 60–90% of the carbon into the Earth’s atmosphere. Clearly, metamorphic CO_2_ production can have a relatively large climatic effect^20,39^.

**Fig. 8:**
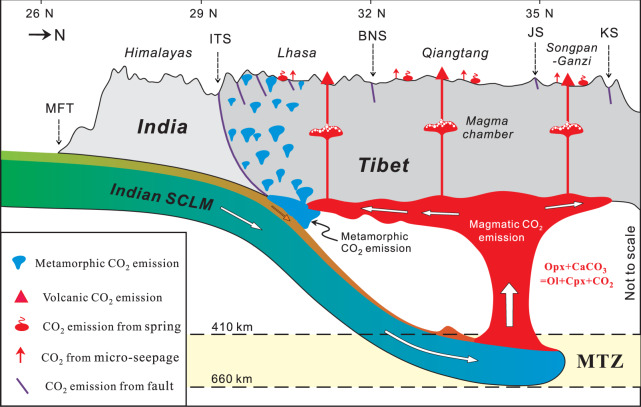
Major types of continental collision-derived CO_2_ degassing, including metamorphic and magmatic CO_2_ emissions in Tibet. Magmatic CO_2_ emissions are composed of Cenozoic volcanic activity-induced CO_2_ emissions and the present CO_2_ degassing from the hydrothermal systems (e.g., hot spring, fault and soil micro-seepage) related to dormant volcanic fields in the Plateau. MFT main frontal thrust, MTZ mantle transition zone, SCLM sub-continental lithospheric mantle. ITS, BNS, JS, and KS are as in Fig. 1.

Stage 2(b) (50–25 Ma) occurred in the climax of Indian subduction (Fig. 2c), resulting in massive enrichment of a carbonate-containing component in the mantle source region of the magmas (Table 1; Supplementary Fig. 6) and a large-scale upwelling of a CMP (Figs. 2 and 8)^16^. Because the Sr–Nd–Pb isotopic compositions of the magmatic rocks (Supplementary Figs. 3 and 6) indicate that the previous deep carbon reservoir involved in the plume-wedge interaction had been fully exhausted by the end of Stage 2(a) (see Eq. (1)), magmatism-related outgassing plays anew a vital role in controlling on the atmospheric CO_2_ variations in Stage 2(b). Our CCFM results (Fig. 7b) indicate declining CO_2_ emission rates from 50 to 25 Ma, which is consistent with waning magmatic activity along the southern Eurasian margin from early to late in Stage 2(b)^8,16,37^. This suggests that magmatism-related outgassing resulted in decreases in the atmospheric CO_2_ levels and resulting climate cooling since ca. 50 Ma^1,2,8^. In contrast, in Stage 2(b), metamorphic outgassing in Tibet no longer plays a role in controlling global atmospheric CO_2_ variations.

Stage 3 (25–0 Ma) resulted from India and Asia continental subduction because both India- and Asia-derived silicate-rich components have been identified based on their Sr–Nd–Pb isotope compositions (Supplementary Fig. 6, Table 2). The mantle source region of the Stage 3 magmas has a much higher concentration of the silicate-rich component than that in Stage 2 (Table 1 and Fig. 3). This suggests that the carbonate platforms and carbonate-rich basins on the northern margin of India were fully subducted and recycled within the mantle wedge by the end of Stage 2, and thus India- and Asia-derived, silicate-rich continental material entered the subduction zone thereafter (Table 2; Supplementary Fig. 6). This has thus resulted in the occurrence of a dual continental convergence, subduction-induced (Fig. 2d), intermediate CO_2_ degassing flux during Stage 3 (Table 1; Fig. 7b), causing low atmospheric CO_2_ levels from 25 to 0 Ma (Fig. 7a). A lack of magmatism from ~8 Ma to the present in south Tibet^14–16,41^ corresponds to a further decrease of the CO_2_ outgassing flux (Table 1; Fig. 7b), which possibly led to late-Miocene climate cooling^3^.

We thus propose that three-stage subduction led to two stepwise transformations of magmatism and mantle degassing in Tibet, namely the transformations in CO_2_ degassing mechanism at 55 Ma and the deep carbon reservoir at 25 Ma (Figs. 2 and 7b). This may have resulted in global palaeoclimate evolution from greenhouse to icehouse conditions (Fig. 7a), suggesting a vital deep carbon cycle process in the Cenozoic.

It should be noted that previous studies^42–45^ have indeed proposed that CO_2_ emissions from Cenozoic continental arc volcanism, mainly composed of Neo-Tethyan and/or Pacific Ocean lithosphere subduction-related volcanic activity, has the potential to control global atmospheric CO_2_ concentration variations and palaeoclimate change since ~65 Ma. However, here we distinguish clearly and fundamentally between the India-Asia continent collision-derived CO_2_ degassing of Stages 2–3 (55–0 Ma) and the Neo-Tethyan Ocean lithosphere subduction-related CO_2_ emissions of Stage 1 (65–55 Ma). These distinctions are based on (a) the magmatic rock types (Fig. 1), geochemical compositions (Supplementary Data 2; Figs. 3 and 4) and petrogenesis (Fig. 2), (b) CO_2_ degassing mechanism and types (Figs. 6 and 7), and (c) magnitudes of magmatic and metamorphic CO_2_ degassing rates (Fig. 7b). These distinctions force us to the conclusion of different contributions from these geodynamic settings (i.e., Neo-Tethyan Ocean lithosphere subduction and India-Asia continent collision) to global carbon outgassing in the Cenozoic.

Based on the compositions and petrogenesis of magmatic rocks (Fig. 2) and the magnitude of CO_2_ outgassing (Fig. 7b), we further propose that deep carbon cycle processes in the Cenozoic may be subdivided into the following two main types: (a) India-Asia continent collision-derived CO_2_ degassing (including Stages 2–3) and (b) ocean lithosphere subduction-related CO_2_ emission (including Neo-Tethyan subduction in Stage 1 and Pacific Ocean lithosphere subduction since 65 Ma). Overall, our study indicates that India-Asia continent collision-derived CO_2_ outgassing may have been a critical driver of the atmospheric CO_2_ concentration variations and palaeoclimatic changes in the Cenozoic, as it displays much higher CO_2_ degassing flux than that of ocean lithosphere subduction (Fig. 7b). In detail, our three-component mixing model calculation results (Table 1 and Fig. 3) from the Sr–Nd–Pb isotope compositions indicate that India continent subduction has resulted in a greater proportion (0.97%) of recycled carbonate sediments in the source region of the magmas in Stage 2 (55–25 Ma) than is the case for Neo-Tethyan (0.08%) in Stage 1 (65–55 Ma) and Pacific^46,47^ Ocean lithosphere subduction in the Cenozoic. The CCFM calculations (Table 1 and Fig. 7b) also indicate that the India-Asia collision-derived CO_2_ fluxes (0.0838–6.7206 Pg/year) in Stage 2 (55–25 Ma) are two orders of magnitude higher than the magmatic CO_2_ fluxes degassed from Neo-Tethyan (0.0002–0.0035 Pg/year) in Stage 1 (65–55 Ma) and Pacific Ocean^43–45^ lithosphere subduction. Previous studies^48,49^ have indicated that the oceanic lithosphere subduction-derived CO_2_ rate is constant during the Cenozoic, which cannot alone generate an observed three-stages of atmospheric CO_2_ concentration variations. Thus, despite the fact that Pacific Ocean lithosphere subduction-related volcanism is extensive from 65 Ma to the present^43,50^ we infer the dominance of an India-Asia collision-derived CO_2_ flux over ocean subduction-derived CO_2_ flux in the Cenozoic, together with a correspondingly minor contribution of Neo-Tethys and Pacific Ocean lithosphere subduction-induced arc volcanic CO_2_ degassing to Cenozoic climate changes.

Magmatic carbon emission is mainly composed of (1) CO_2_ degassing during the active volcanic period and (2) CO_2_ emissions from diffuse faults, hot springs and soil micro-seepage systems during dormant volcanic periods^43,45^. The near-continuous time-series of modelled CO_2_ fluxes from eruptions of 55 volcanic fields during the Cenozoic in Tibet (Table 1 and Fig. 7b) reveal CO_2_ degassing during active volcanic periods from 65 Ma to the present. Today, large-scale CO_2_ emissions from hot springs, geysers, steaming fissures, diffuse faults (and rifts) and soil CO_2_ micro-seepage fields are widely distributed in the Tibetan Plateau^45,51^ and are considered to be one of the most important hydrothermal systems in dormant volcanic fields related to deep Earth-derived CO_2_ degassing in the world^45,52^. Extensive field measurements indicate that the current CO_2_ flux degassed from these dormant volcanic fields is ca. 15 Mt/year in Tibet, in agreement with our CCFM calculations (i.e., 17–20 Mt) (Fig. 7b). He and C isotope studies of CO_2_ emitted from the hot springs and soil CO_2_ micro-seepage related to these dormant volcanic fields are consistent with our interpretation as subducted India-derived deep carbon emissions within the rifts and geothermal systems in the Tibetan Plateau^45,51,53^. Three-component mixing model calculations of the Sr–Nd–Pb isotope compositions of the Cenozoic magmatic rocks in Tibet (Tables 1 and 2) are also consistent with an India-derived carbonate-rich component having been subducted into the upper mantle beneath the Plateau, which has resulted in volcanic CO_2_ emissions during the eruptions of the volcanic fields in the Tibetan Plateau (Figs. 1 and 5). One might ask how the India subduction-derived carbonate-rich component was transformed into the various surface forms of magmatic CO_2_ emissions (including above mentioned volcanic activity-induced CO_2_ emissions and passive volcanic CO_2_ emissions; Fig. 8) in the Tibetan Plateau during the Cenozoic? An answer might lie in the possibility that the India subduction-derived carbonate (e.g., dolomite)-rich component (Table 2 and Fig. 2) was capable of metasomatizing lherzolites and harzburgites to yield wehrlite-bearing mantle peridotite within the mantle wedge beneath Tibet (Fig. 8)^16^ with the result that CO_2_ output from decarbonation reaction (Eq. (3))^54,55^ through CMP upwelling^16,56^ could serve as the main mechanism of the current surface forms of magmatic CO_2_ degassing.

3 $${\rm{2Mg}}_{2}{\rm{Si}}_{2}{\rm{O}}_{6}({\rm{enstatite}}) 	+{\rm{CaMg}}({{\rm{CO}}}_{3})_{2}({\rm{dolomite}})\to {\rm{2Mg}}_{2}{\rm{SiO}}_{4}({\rm{olivine}})\\ 	 +{\rm{CaMgSi}}_{2}{\rm{O}}_{6}({\rm{diopside}})+2{\rm{CO}}_{2}({\rm{vapour}})$$

where Mg_2_Si_2_O_6_ denotes orthopyroxene, enriched in lherzolites and harzburgites in the upper mantle beneath the Tibetan Plateau. CaMg(CO_3_)_2_ refers to India continent subduction-derived carbonate-rich component. Mg_2_SiO_4_ + CaMgSi_2_O_6_ represents an olivine and clinopyroxene assemblage enriched in wehrlites within the metasomatised mantle wedge beneath the Tibetan Plateau and neighbouring areas, which has been corroborated by the recent petrologic and geochemical studies (e.g., ref. ^16^). CO_2_ means magmatic CO_2_ emission mainly including (1) Cenozoic volcanic activity-induced CO_2_ degassing and (2) CO_2_ emissions from the diffuse faults, hot springs and soil micro-seepage systems related to dormant volcanic fields in the Tibetan Plateau at the present.

Subduction of the Indian continent may have resulted in the occurrence of carbonatitic metasomatism in the upper mantle beneath the Plateau (Table 1; Figs. 3 and 8), leading to upwelling of a CMP below Tibet (Figs. 2c and 8)^16,56^. The CMP upwelling would cause continuous exsolution of CO_2_^57^, which could lead to further continent subduction-derived CO_2_ degassing in Tibet in the Cenozoic (Fig. 7b). We thus propose that the *ca.* 3000 km-long subducting Indian continental lithospheres could play a significant role in controlling the Cenozoic CO_2_ emission rates in Tibet, based on a conservative value of 5 cm/year for the convergence rate since India-Asia collision at ~60 Ma^58,59^.

It should be noted that there is an inconsistency between CO_2_ degassing flux and atmospheric CO_2_ concentrations in Stages 1 and 3, namely the occurrence of a low CO_2_ flux resulted in the intermediate atmospheric CO_2_ levels in Stage 1, whereas the occurrence of an intermediate CO_2_ flux led to low atmospheric CO_2_ levels in Stage 3 (Fig. 7). A lack of quantitative calculations of net CO_2_ contributions based on comparisons between CO_2_ production rate from deep Earth degassing (i.e., magmatism and metamorphism) and CO_2_ consumption rate from silicate weathering in Stages 1 and 3 make this aspect difficult to explain further at this time. Recently, Botsyun et al.^60^ have proposed that revised paleoaltimetry data indicate a low elevation during the Eocene and probable strong late-Cenozoic uplift in the Tibetan Plateau. This may indicate an important role for strong chemical weathering from the following reaction Eq. (4)^4,9^ due to the strong late-Cenozoic uplift in Tibet^60,61^, possibly in turn leading to strong declines in global atmospheric CO_2_ levels in Stage 3.

4 $${{\rm{CaSiO}}}_{3}+{{\rm{CO}}}_{2}\to {{\rm{CaCO}}}_{3}+{{\rm{SiO}}}_{2}$$

Without a doubt, the timing of the initiation of Tibetan uplift remains controversial^60–62^. In the context of this contribution, this aspect of the problem is worthy in future of more in-depth analysis and discussion.

We conclude that deep earth degassing has played a critical role in controlling global atmospheric CO_2_ concentration variations and palaeoclimate changes during the Cenozoic, implying an integrated system of continental collision-related deep carbon cycle processes. This view of Earth degassing includes primarily magmatic and metamorphic CO_2_ emissions, whereby the flux of metamorphic CO_2_ outgassing is much higher than that of magmatism. Despite the fact that both resulted from Neo-Tethys and subsequent Indian continent subduction the mechanisms of metamorphic and magmatic CO_2_ outgassing are quite distinct. The links between geochemical modelling calculations and the deep carbon cycle employed here have the potential to become a significant new application of future studies of major element, trace element and isotopic data for magmatic rocks.

## Methods

### Calculation approaches of the CCFM

We developed a model to evaluate how much CO_2_ could have been released during the India-Asia collision, based on calculations of mean values of eruptive ages in the different volcanic fields and geochemical modelling calculations, using geochronological (Supplementary Data 1) and geochemical (Supplementary Data 2) data for the magmatic rocks in the Tibetan Plateau throughout the Cenozoic. Detailed calculation methods and processes of the CCFM include the following components:

### Calculation methods of mean values of eruptive ages in the different volcanic fields

We first compiled previously published geochronological data for the magmatic rocks in the Tibetan Plateau (Supplementary Data 1). The results of some dating approaches (e.g., whole-rock K–Ar dating without errors) have been excluded due to their poor quality^14–16^. The previous sampling locations were then checked in order that amounts of the geochronological data are broadly consistent with areas of outcrops of the magmatic rocks in each volcanic field in Tibet. The magnitude of the magmatism is considered to be a normal distribution from early to late in each volcanic field because it results from partial melting of enriched upper mantle involved in India-Asia collision^14–16,33^. Thus, based on a normal distribution of the geochronological data in each volcanic field of the Tibetan Plateau, a mean value (*M*) and standard deviation (SD) may be calculated by Eq. (5A-5B)^63^:

5A $${{M}}=\frac{{\sum }_{i=1}^{n}X}{n}$$

5B $${\rm{SD}}=\sqrt{\frac{\sum {(X-M)}^{2}}{n-1}}$$

where *X* denotes the individual data points in each volcanic field. Σ (sigma) refers to the sum for all the *n* data points in each volcanic field of the Tibetan Plateau. Our calculation results for the mean values and standard deviations are shown in Table 1 and Supplementary Data 1.

### Determination methods of compositions of the subducting materials and source regions of magmas

Mixing theory of radiogenic isotopes^35^ indicates that the isotopic ratios of three-component mixtures plot within a compositional triangle which is defined by the three-endmembers in correlation diagrams of the isotope ratios. Based on the above rationale, we firstly plotted the Sr–Nd–Pb isotope ratios of the magmatic rocks in Tibet and thus determined their three-component mixing characteristics in the mantle source regions of the magmas, because the Tibetan magmatic rocks plot within compositional triangles in these isotope correlation diagrams (Supplementary Figs. 3 and 6). We then determined the compositions of the end-members of the subducting materials (Table 2) through recognition of the subduction-derived metasomatic agents in the three-component mixtures, because the Tibetan magmatic rocks resulted from Neo-Tethys and Indian subduction in the Cenozoic^14–16,33^. In addition, amounts of the carbonate-rich components in the subducting materials, which could be recycled via magmatic CO_2_ degassing in Tibet, were estimated in the following geochemical model calculations.

### Calculation methods of proportions of end-members in the source region of the magmas

On the basis of the determinations of the compositions of three end-members in the mantle source regions of the Cenozoic magmatic rocks in Tibet (Table 2), we performed three-component mixing model calculations of the Sr–Nd–Pb isotope compositions of the three-stage magmas according to the rationale of mixing theory^35,36^. The detailed calculation procedures are as follows. The mixing theory of radiogenic isotopes^35^ indicates that three-component mixing model calculations of the isotope compositions can be treated like a couple of the two-component mixing calculations. Namely, we may firstly carry out a two-component mixing model calculation, then we may use this two-component mixing calculation result to mix with a third component^35^. The two-step two-component mixing processes would finally yield the three-component mixing model calculations for the isotope compositions^35^.

According to the above two-step mixing calculation method (e.g., Sr and Nd isotopes), proportions of the subducted silicate sediments (Δ*s*) in the three-component mixing mantle source region of the Tibetan magmatic rocks may be calculated as follows. We firstly carried out a two-component mixing model calculation between the MORB-source mantle and the carbonate sediments using Eq. (5C-5F)^35^.

5C $${({\,}^{87}{\rm{Sr}}/{\,}^{86}{\rm{Sr}})}_{2{\rm{M}}}={({\,}^{87}{\rm{Sr}}/{\,}^{86}{\rm{Sr}})}_{\rm{c}}(\varphi )({\rm{Sr}}_{c}/{\rm{Sr}}_{2M})+{({\,}^{87}{\rm{Sr}}/{\,}^{86}{\rm{Sr}})}_{B}(1-\varphi )({\rm{Sr}}_{B}/{\rm{Sr}}_{2M})$$

5D $${\rm{Sr}}_{2M}={\rm{Sr}}_{c}\times \varphi +{\rm{Sr}}_{B}\times (1-\varphi)$$

5E $${({\,}^{143}{\rm{Nd}}/{\,}^{144}{\rm{Nd}})}_{2{\rm{M}}}={({\,}^{143}{\rm{Nd}}/{\,}^{144}{\rm{Nd}})}_{\rm{c}}\varphi ({\rm{Nd}}_{c}/{\rm{Nd}}_{2M})+{({\,}^{143}{\rm{Nd}}/{\,}^{144}{\rm{Nd}})}_{B}(1-\varphi )({\rm{Nd}}_{B}/{\rm{Nd}}_{2M})$$

5F $${\rm{Nd}}_{2M}={\rm{Nd}}_{c}\times \varphi +{\rm{Nd}}_{B}\times (1-\varphi )$$

where (^87^Sr/^86^Sr)_c_, (^87^Sr/^86^Sr)_B_ and (^87^Sr/^86^Sr)_2M_ are ^87^Sr/^86^Sr of the carbonate sediments, MORB-source mantle and a two-component mixture between MORB-source mantle and the carbonate sediments, respectively. Sr_c_, Sr_B_, and Sr_2M_ are concentrations of Sr in the carbonate sediments, MORB-source mantle and the two-component mixture between MORB-source mantle and the carbonate sediments, respectively. (^143^Nd/^144^Nd)_c_, (^143^Nd/^144^Nd)_B_, and (^143^Nd/^144^Nd)_2M_ are ^143^Nd/^144^Nd of the carbonate sediments, MORB-source mantle and the two-component mixture between MORB-source mantle and the carbonate sediments, respectively. Nd_c_, Nd_B_ and Nd_2M_ are concentrations of Nd in the carbonate sediments, MORB-source mantle and the two-component mixture between MORB-source mantle and the carbonate sediments, respectively. φ are proportions of the carbonate sediments in the two-component mixture between the MORB-source mantle and the carbonate sediments.

We then performed a final three-component mixing calculation between the above calculated two-component mixture and the silicate sediments using Eq. (5G-5J)^35^.

5G $${({\,}^{87}{\rm{Sr}}/{\,}^{86}{\rm{Sr}})}_{3{\rm{M}}}={({\,}^{87}{\rm{Sr}}/{\,}^{86}{\rm{Sr}})}_{\rm{s}}\varDelta s({\rm{Sr}}_{s}/{\rm{Sr}}_{3M})+{({\,}^{87}{\rm{Sr}}/{\,}^{86}{\rm{Sr}})}_{2M}(1-\varDelta s)({\rm{Sr}}_{2M}/{\rm{Sr}}_{3M})$$

5H $${\rm{Sr}}_{3M}={\rm{Sr}}_{s}\times \varDelta s+{\rm{Sr}}_{2M}\times (1-\varDelta s)$$

5I $${({\,}^{143}{\rm{Nd}}/{\,}^{144}{\rm{Nd}})}_{3{\rm{M}}}={({\,}^{143}{\rm{Nd}}/{\,}^{144}{\rm{Nd}})}_{s}(\varDelta s)({\rm{Nd}}_{\rm{s}}/{\rm{Nd}}_{3M})+{({\,}^{143}{\rm{Nd}}/{\,}^{144}{\rm{Nd}})}_{2M}(1-\varDelta s)({\rm{Nd}}_{2M}/{\rm{Nd}}_{3M})$$

5J $${\rm{Nd}}_{3M}={\rm{Nd}}_{s}\times \varDelta s+{\rm{Nd}}_{2M}(1-\varDelta s)$$

where (^87^Sr/^86^Sr)_s_, (^87^Sr/^86^Sr)_2M_ and (^87^Sr/^86^Sr)_3M_ are ^87^Sr/^86^Sr of the silicate sediments, the above calculated two-component mixture between MORB-source mantle and the carbonate sediments and the final three-component mixture between the above two-component mixture and the silicate sediments, respectively. Sr_s_, Sr_2M_ and Sr_3M_ are concentrations of Sr in the silicate sediments, the above calculated two-component mixture between MORB-source mantle and the carbonate sediments and the final three-component mixture between the above two-component mixture and the silicate sediments, respectively. (^143^Nd/^144^Nd)_s_, (^143^Nd/^144^Nd)_2M_ and (^143^Nd/^144^Nd)_3M_ are ^143^Nd/^144^Nd of the silicate sediments, the above calculated two-component mixture between MORB-source mantle and the carbonate sediments and the final three-component mixture between the above two-component mixture and the silicate sediments, respectively. Nd_s_, Nd_2M_ and Nd_3M_ are concentrations of Nd in the silicate sediments, the above calculated two-component mixture between MORB-source mantle and the carbonate sediments and the final three-component mixture between the above two-component mixture and the silicate sediments, respectively.

Following the above isotope mixing model calculation processes, we first carried out a two-component mixing calculation between the MORB-source mantle and the subducted silicate sediments and then performed a three-component mixing calculation among the MORB-source mantle, the subducted silicate and carbonate sediments. We finally obtain proportions of the subducted carbonate sediments (Δ*c*) in the three-component mixtures between MORB-source mantle, the subducted silicate and carbonate sediments. The three-component mixing model calculation results are shown in Table 1 and Supplementary Data 2.

On the basis of determinations of the proportions of metasomatic components derived from subducted silicates and subducted carbonates in the mantle source of the three-stage magmatic rocks (Table 1), we calculated the degree of partial melting (*ϕ*_*f*_) and proportions of the residual minerals (*X*_*i*_) in the mantle source region of the three-stage Tibetan magmas using trace element contents in the magmatic rocks through a non-modal batch melting model^36^. The modelling rationale and approach of this quantitative calculation in detail follow those of ref. ^36^. The calculated formulae for the degree of partial melting (*ϕ*_*f*_) and proportions of the residual minerals in the mantle source region of the Tibetan magmas (*X*_*i*_) are shown in Eq. (5K-5M)^36^:

5K $$D=\sum {X}_{i}{D}_{i}$$

5L $$P=\sum {P}_{i}{D}_{i}$$

5M $${C}_{L}/{C}_{o}=1/({\phi }_{f}+D-{\phi }_{f}P)$$

where *D* is the bulk distribution coefficient. *D*_*i*_ is the crystal-liquid partition coefficient of phase *i* in the mineral assemblage. *P*_*i*_ is the proportion of phase *i* entering the melt. *C*_*L*_ is the concentration of a trace element in the melt. *C*_*o*_ is the initial concentration of a trace element in the metasomatised mantle source, which is thought to be the three-component mixture^36^.

The approach and procedure of calculation of the above non-modal batch melting model are as follows. We selected two initial parameters in the modelling calculations of the non-modal batch melting equations in the first step^36^; these are (1) the partial melting degree and (2) the residual mineral proportions in the mantle source region. We also selected a potential range for the melting degree from 0 to 30% with a calculated step of 1% based on the previously published results for the melting degree required for Cenozoic magma generation in Tibet^14–16,33^ in the second step. Considering the presence of new mineral phases in the source region (e.g., phlogopite, amphibole and apatite) formed during metasomatism of the mantle wedge, we estimated that the resultant mineral modes (and the potential ranges in their proportions) in the enriched mantle caused by infiltration of the subduction-derived component were: olivine (from 10 to 60%), orthopyroxene (from 5 to 60%), clinopyroxene (from 5 to 60%), spinel (from 0 to 10%), phlogopite (from 0 to 15%), amphibole (from 0 to 20%), rutile (from 0 to 10%), titanite (from 0 to 20%) and apatite (from 0 to 10%), based on previous melting experiments and model calculations^14–16,33,41^.

On the basis of the above ranges in the initial parameters, we carried out the non-modal batch melting model calculations by changing the parameters from the minimum to maximum values within their respective potential ranges. When the modelling calculation results provided the best fit to the actual trace element concentrations in the Cenozoic magmatic rocks, we terminated the calculation and recorded the final values of the trace element concentrations, residual mineral proportions and melting degree of the mantle source region. Thus, the final calculation results of our trace element melting model are values of the degree of partial melting (*ϕ*_*f*_) and proportions of the residual minerals (*X*_*i*_) in the mantle source region of the three-stage magmas in Tibet (Table 1). If there are more than one combination of trace element contents that would minimise the misfit between analytical data and model predictions, we finally select and determine the model calculation results through (1) geologically reasonable judgement of the calculated values of the degree of partial melting (*ϕ*_*f*_) and proportions of the residual minerals (*X*_*i*_) in the mantle source region of the three-stage magmas in Tibet and/or (2) calculations of average values of above those parameters.

The final aim of the geochemical modelling calculations (Table 1 and Supplementary Data 2) is to estimate values of four geochemical parameters, which include the proportions of the subducted silicate (Δ*s*) and subducted carbonate (Δ*c*) sediments, and the degree of partial melting (*ϕ*_*f*_) and proportions of the residual minerals (*X*_*i*_) in the mantle source region of the magmas. We thus may obtain a mean value (Table 1) of the above four geochemical parameters for each volcanic field of the Tibetan Plateau using new and previously published geochemical data for the Cenozoic magmatic rocks (Supplementary Data 2), based on a normal distribution of the data using the above formula (5). These geochemical modelling calculation results (Table 1) are the important input parameters for the estimates of the collision-induced CO_2_ degassing flux in subsequent CCFM calculations. The proportions of the subducted carbonate sediments (Δ*c*), the proportions of the subducted silicate sediments (Δ*s*) and the degrees of partial melting (*ϕ*_*f*_) are required to calculate the magmatic CO_2_ degassing rate in the continental collision, whereas the proportions of the residual minerals (*X*_*i*_) in the mantle source region of the magmas are important parameters to calculate the metamorphic CO_2_ degassing rate in the continental collision.

### Calculation methods of magmatic CO_2_ degassing flux in continental collision

The proportion of the carbonate-rich component in the source region of the magmatic rocks (Δ*c*) refers to how much of the subducted carbonate is transported to the mantle wedge beneath the Tibetan Plateau, which is an important deep carbon source for magmatic (including volcanic) CO_2_ degassing in the continental collision. The melting degree in the source region of the magmas (*ϕ*_*f*_) describes the proportion of partial melting of the carbonate-rich mantle wedge, which denotes how much of the subducted carbon is transported back to the atmosphere through Cenozoic magmatism in Tibet. A product of Δ*c* and *ϕ*_*f*_ could be used to calculate the recycling efficiency of subducted carbonate during the continental collision-induced deep carbon cycle, based on the rationale of partial melting of the mantle wedge and its metasomatism^35,36^. Thus, the recycling efficiency (*f*_*e*_), which refers to the proportion of recycled carbon during the whole cycle from Neo-Tethyan oceanic to Indian continental subduction in the Cenozoic, can be defined by Eq. (6):

6 $${f}_{e}=\left(\frac{{\varDelta }_{c}}{{\varDelta }_{c}+{\Delta }_{s}}\right)\times {\phi }_{f}$$

where 1/(Δ*c* + Δ*s*) refers to a transfer coefficient from a three-component (carbonate- and silicate-rich components, MORB-source mantle) mixing mantle source region of the magmas to a two-component (carbonate- and silicate-rich components) composing subducted lithospheric slabs (Table 2) because the component of MORB-source mantle in the mantle source region of the magmas is not involved in the recycling processes. Thus, values of the parameters of Δ*c* in the three-component mixing mantle source region of the magmas would be transferred into that in the two-component model comprising subducted carbonates and silicates, multiplied by a ratio of 1/(Δ*c* + Δ*s*). The calculated results of the recycling efficiency (*f*_*e*_) in the different volcanic fields in Tibet are shown in Table 1 and Supplementary Data 2.

Based on the above calculation of the recycling efficiency (*f*_*e*_), following the formula [S2] in Jagoutz et al.^19^, the rate of magmatic CO_2_ degassing into the atmosphere (*u*_*e*_) is given by Eq. (7):

7 $${u}_{e}={\gamma }_{t}\times {\delta }_{t}\times {\mu }_{t}\times \rho \times {f}_{e}$$

where γ_*t*_ and *δ*_*t*_ are subducted slab thickness and width parallel to the trench in the whole Cenozoic through time, respectively. They are taken from Jagoutz et al.^19,22^ and Shi et al.^56^. *μ*_*t*_ is the India-Asia convergence rate in the Cenozoic through time, which is taken from Jagoutz et al.^22^ and Lee and Lawver.^64^. *ρ* is a constant, which is taken from ref. ^65^. Our calculation results of the magmatic CO_2_ degassing rate are shown in Table 1 and Fig. 6.

### Calculation methods of metamorphic CO_2_ outgassing rate in continental collision

Sr–Nd–Pb isotope compositions determine that a plume-wedge interaction occurred in Stage 2(a) from 55 to 50 Ma (Supplementary Fig. 3). This interaction would reactivate a long-term hidden huge, deep carbon reservoir in the mantle wedge caused by the previous subduction of the Neo-Tethys Ocean lithosphere (Fig. 8). It may further lead to CO_2_ emitted into the atmosphere through metamorphism by the carbonate dissolution reaction^20,39^, which is displayed as the chemical reaction (Eq. (1)) in the above main text.

In the above carbonate dissolution reaction equation (Eq. (1)) (i.e., CaCO_3_ + 2H^+^ → Ca^2+^ + H_2_O + CO_2_), CaCO_3_ represents a carbon reservoir previously preserved in the mantle wedge beneath Tibet (Fig. 8). H^+^ denotes an H^+^-rich component in the upwelling CMP resulted from the presence of new mineral phases in the source region of the magmas (e.g., phlogopite and amphibole) formed during metasomatism of the mantle wedge (Fig. 8), which can be estimated using the proportions of the water (i.e., OH^−^)-containing residual minerals in the source region of the Tibetan magmas (*X*_*i*_) (Table 1 and Supplementary Data 2). CO_2_ refers to metamorphic CO_2_ degassing. A transfer coefficient (*d*) from a reactant (i.e., H^+^) to a product (i.e., metamorphic CO_2_) in the carbonate dissolution reaction (Eq. (1)) can be defined by Eq. (8)^20,39^:

8 $$d=\frac{\left[{\rm{CO}}_{2}\right]}{2\left[{\rm{H}}\right]}$$

where [CO_2_] and [H] represents the molar mass of CO_2_ and H, respectively.

According to modelling of recycling processes of the enriched components in the convection system of the mantle wedge within a subduction zone (Fig. 5)^66,67^, following the formula [S2] in Jagoutz et al.^19^, the mass flow rate (*S*_*e*_) of the uprising transportation of the H^+^-rich component in upwelling of the CMP may be calculated by^20,39^:

9 $${S}_{e}={\partial }_{t}\times {\delta }_{t}\times {\omega }_{t}\times \rho \times {H}_{i}^{+}$$

where ${H}_{i}^{+}$ is taken from Table 1 and Supplementary Data 2. ∂_*t*_ denotes the width of the spatial distribution of the Tibetan magmatic rocks during Stage 2(a) in the direction perpendicular to the trench through time (Fig. 1). ω_*t*_ is the upwelling rate of CMP in the Cenozoic through time, which is estimated by the formula [4] in Soltanmohammadi et al.^66^. Other parameters are as in Eq. (7). The mass flow rate (*S*_*e*_) refers to the flux of the H^+^-rich component by upwelling of the CMP based on the carbonate dissolution reaction (Eq. (1)) in Stage 2(a).

According to the above carbonate dissolution reaction (Eq. (1)), a product of *S*_*e*_ and *d* could be used to calculate the metamorphic CO_2_ degassing flux (*m*_*e*_)^20,39^. Thus, based on the above Eqs. (8) and (9), the metamorphic CO_2_ degassing flux (*m*_*e*_) may be defined by Eq. (10)^20,39^:

10 $${m}_{e}={S}_{e}\times d$$

The calculation results of the metamorphic CO_2_ degassing flux are shown in Table 1 and Fig. 6.

### Calculation methods of continental collision-induced CO_2_ degassing rate in Tibet

Based on the above we computed the sum of the magmatic and metamorphic CO_2_ degassing rates in each volcanic field from 65 Ma to the present, which display the temporal variations of collision-induced CO_2_ degassing rate with the mean ages of the different Tibetan volcanic fields in the Cenozoic (Fig. 7b). The detailed calculated procedure of the CCFM follows those formula step by step in Supplementary Data 2.

### Calculation methods of uncertainties of results of CCFM

The standard deviation for the collision-induced CO_2_ degassing rates reflects the range of the CO_2_ degassing rate values for the different Tibetan volcanic fields in the Cenozoic (Supplementary Data 2), which resulted from the propagated uncertainty associated with those of the model input initial parameters (i.e., standard deviation) including in Δ*c*, Δ*s*, *ϕ*_*f*_ and *X*_*i*_ at each volcanic field in Tibet (Supplementary Data 2). The above-modelled calculation processes indicate that these initial parameters contributed roughly equally to the uncertainty because the CCFM is a fully forward model and all equations are analytical solutions. For figure legibility, instead of the error bars, we have shown a curve of the mean value for our predicted Cenozoic CO_2_ history using smoothed fits of the mean value data in each volcanic field over the Cenozoic (Fig. 7b).

### The methods of the whole-rock major and trace element and Sr–Nd–Pb isotope analyses

Detailed sample preparation and analytical procedures for the whole-rock major element, trace element, and Sr–Nd–Pb isotopic analyses are described in Guo et al.^33^ and Guo et al.^68^ and are briefly summarised below. The whole-rock geochemical analyses (Supplementary Data 2) were performed at the Institute of Geology and Geophysics, Chinese Academy of Sciences (IGGCAS), Beijing, China.

Whole-rock samples were trimmed of any altered surfaces and then sawn into thin slices (6 cm). Polished thin sections were prepared from 1 slice and investigated via optical microscopy to confirm the absence of hydrothermal alteration. The remaining slices were cleaned, dried, crushed, and finally ground to a powder using an agate mill. These powders were used for whole-rock major element, trace element, and Sr–Nd–Pb isotopic analysis.

Whole-rock major element compositions were determined using X-ray fluorescence spectrometry. Whole-rock sample powders (0.6 g) were fused with Li_2_B_4_O_7_ (6 g) at 1100 °C for 10 min to produce homogeneous glass pellets for analysis. The analytical precision determined based on two standards was better than 2%. Loss on ignition was determined on 2 g splits of whole-rock powders, which were held at 1100 °C for 10 h.

Rare-earth element and trace element concentrations were determined by inductively coupled plasma mass spectrometry (ICP-MS) on whole-rock samples after their acid digestion in high-temperature Teflon screw-cap capsules. The solutions were diluted up to 50 ml in 1% HNO_3_ for analysis by ICP-MS. A blank solution was prepared and the total procedural blanks were <50 ng for all the trace elements. Analyses of the international rock standards (BHVO-1 and AGV-1) confirm the accuracy of better than 5% for trace and rare earth elements.

Whole-rock Sr–Nd isotope compositions were analyzed using a Finnigan MAT262 mass spectrometer. Sample powders (60 mg) were spiked with mixed isotope tracers (^87^Rb–^84^Sr for Rb–Sr isotope analysis and ^149^Sm–^150^Nd for Sm–Nd isotope analysis), dissolved with a mixed acid (HF: HClO_4_ = 3:1) in Teflon capsules for 7 days at room temperature, and then separated by a cation exchange technique. The collected Sr and Nd fractions were evaporated and dissolved in 2% HNO_3_ to generate solutions for analysis. The mass fractionation corrections for Sr and Nd isotope ratios were based on ^86^Sr/^88^Sr = 0.1194 and ^146^Nd/^144^Nd = 0.7219, respectively. The analyzed procedure blank is less than 2 × 10^−10^ g for Rb–Sr isotope analysis and 5 × 10^−11^ g for Sm–Nd isotope analysis. The ^87^Rb/^86^Sr and ^147^Sm/^144^Nd ratios were calculated using the Rb, Sr, Sm, and Nd contents analyzed by ICP-MS.

Whole-rock Pb isotope ratios were analyzed using a VG354 mass spectrometer. Whole-rock sample powders (150 mg) were dissolved in Teflon capsules using concentrated HF at 120 °C for 7 days. Pb was separated and purified by the AG1 × 8 anion exchange technique. The analyzed blank for this procedure is less than 1 ng. Pb isotope fractionations were corrected for using the factors obtained on the standard NBS 981. The average 2σ uncertainties for ^206^Pb/^204^Pb, ^207^Pb/^204^Pb and ^208^Pb/^204^Pb are 0.6%, 0.4% and 0.5% per atomic mass unit, respectively.

## Supplementary information

Supplementary Information

Description of Additional Supplementary Files

Supplementary Data 1

Supplementary Data 2

## Data Availability

The authors declare that all data supporting the findings of this study are available within the Supplementary Data files (i.e., the source data of Figs. 1–8, Tables 1–2, and Supplementary Figs 1–6 are provided in the Supplementary Data 1 and Supplementary Data 2) associated with this paper.

## Code availability

The calculation procedure and formulae of the CCFM in this study may be followed step by step in Supplementary Data 2, which is publicly available from the formula calculations in the EXCEL file. These formulae are taken from the Methods, which enable reproducibility of the modelling results.
