# Supplementary material for: India-Asia collision as a driver of atmospheric CO2 in the Cenozoic
Source: Nat Commun. 2021 Jun 23;12:3891. doi: 10.1038/s41467-021-23772-y (PMC8222363; doi:10.1038/s41467-021-23772-y)
Supplement: Supplementary file 1 — Supplementary Information [file 41467_2021_23772_MOESM1_ESM.pdf]

SUPPLEMENTARY INFORMATION

India-Asia collision as a driver of atmospheric CO_2 in the Cenozoic

Zhengfu Guo[1,2*], Marjorie Wilson[3], Donald B. Dingwell[4] & Jiaqi Liu[1]

[1] Key Laboratory of Cenozoic Geology and Environment, Institute of Geology and Geophysics, Chinese Academy of Sciences (CAS), Beijing 100029, China

[2] CAS Center for Excellence in Life and Paleoenvironment, Beijing 100044, China

[3] School of Earth and Environment, University of Leeds, Leeds LS2 9JT, UK

[4] Department of Earth and Environmental Sciences, Ludwig-Maximilians-Universität, Munich, Germany

*Corresponding author (e-mail: zfguo@mail.iggcas.ac.cn)

Supplementary Figures

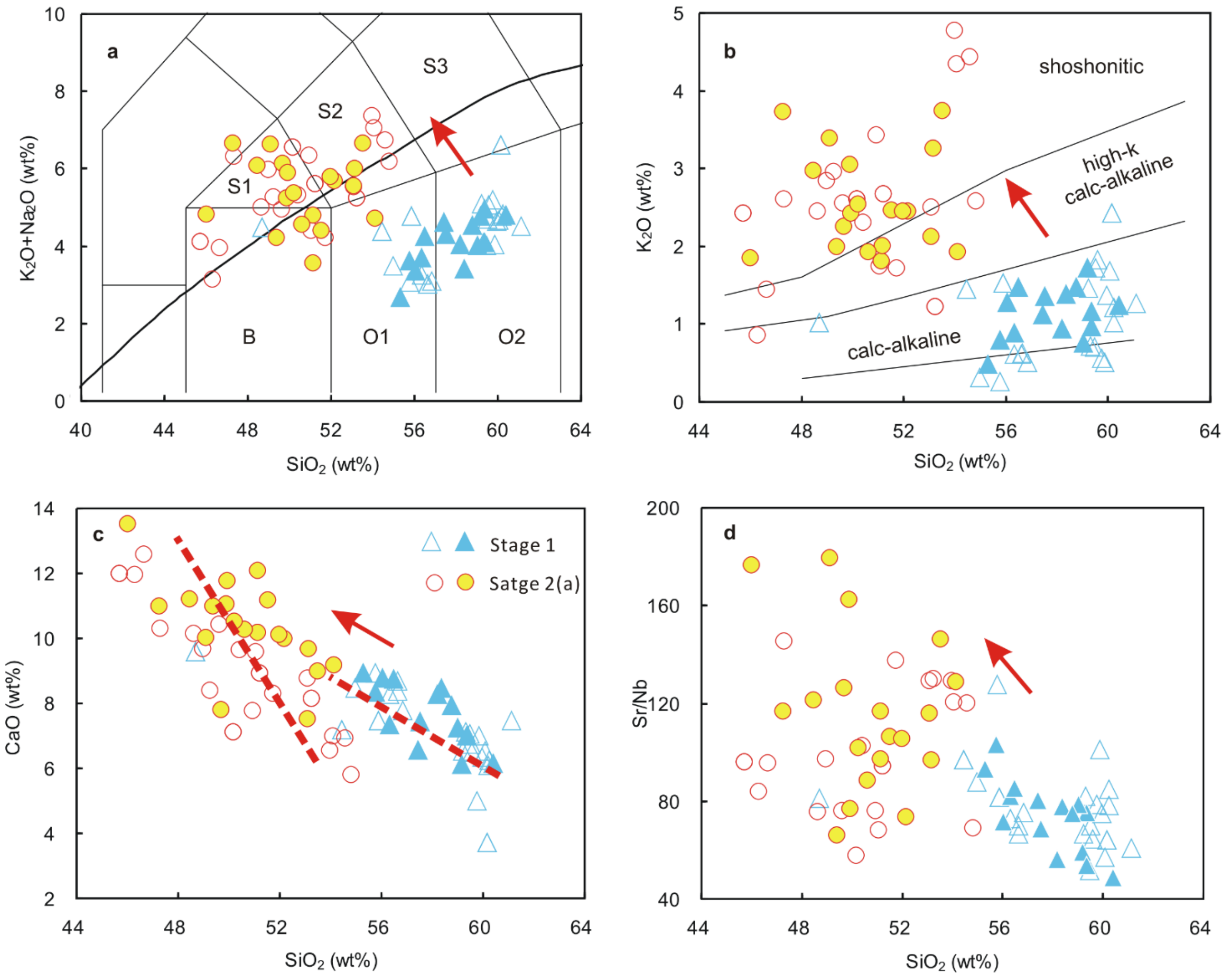

Supplementary Figure 1: Selected major and trace element contents vs SiO_2 (wt %) illustrating silicate-rich metasomatism in the source region of Stage 1 magmas and carbonate-rich metasomatism in the source region of 2(a) magmas. a K_2O+Na_2O (wt%) vs SiO_2 (wt%); **b** K_2O (wt%) vs SiO_2 (wt%); **c** CaO (wt%) vs SiO_2 (wt%); **d** Sr/Nb vs SiO_2 (wt%). Classification boundaries in **a** are from ref.[1]. Rock types in **a** shown by letters are as follows: S1, trachybasalt; S2, basaltic trachyandesite; S3, trachyandesite; B, basalt; O1, basaltic andesite; O2, andesite. The dividing lines in **b** denote the classification boundaries from ref.[2]. Red arrows in **a** to **d** denote the first-step transformation of magmatism at 55 Ma, indicating that Stage 1 magmas result from mantle metasomatism by the silicate-rich component whereas Stage 2 (a) result from metasomatism by the carbonate component, because the former has lower CaO contents and lower Sr/Nb ratios and higher SiO_2 contents than the latter. **a-d** denote increases of the metasomatic carbonate component from Stage 1 to Stage 2 (a). Red dashed lines in **c** represent different trends between CaO (wt%) vs SiO_2 (wt%) in Stage 1 and Stage 2 (a). Filled and open symbols represent, respectively, data from this study (Supplementary Data 2) and the published data[3-10]. The spatio-temporal distributions of Stage 1 and 2 (a) rocks are as in Fig. 1.

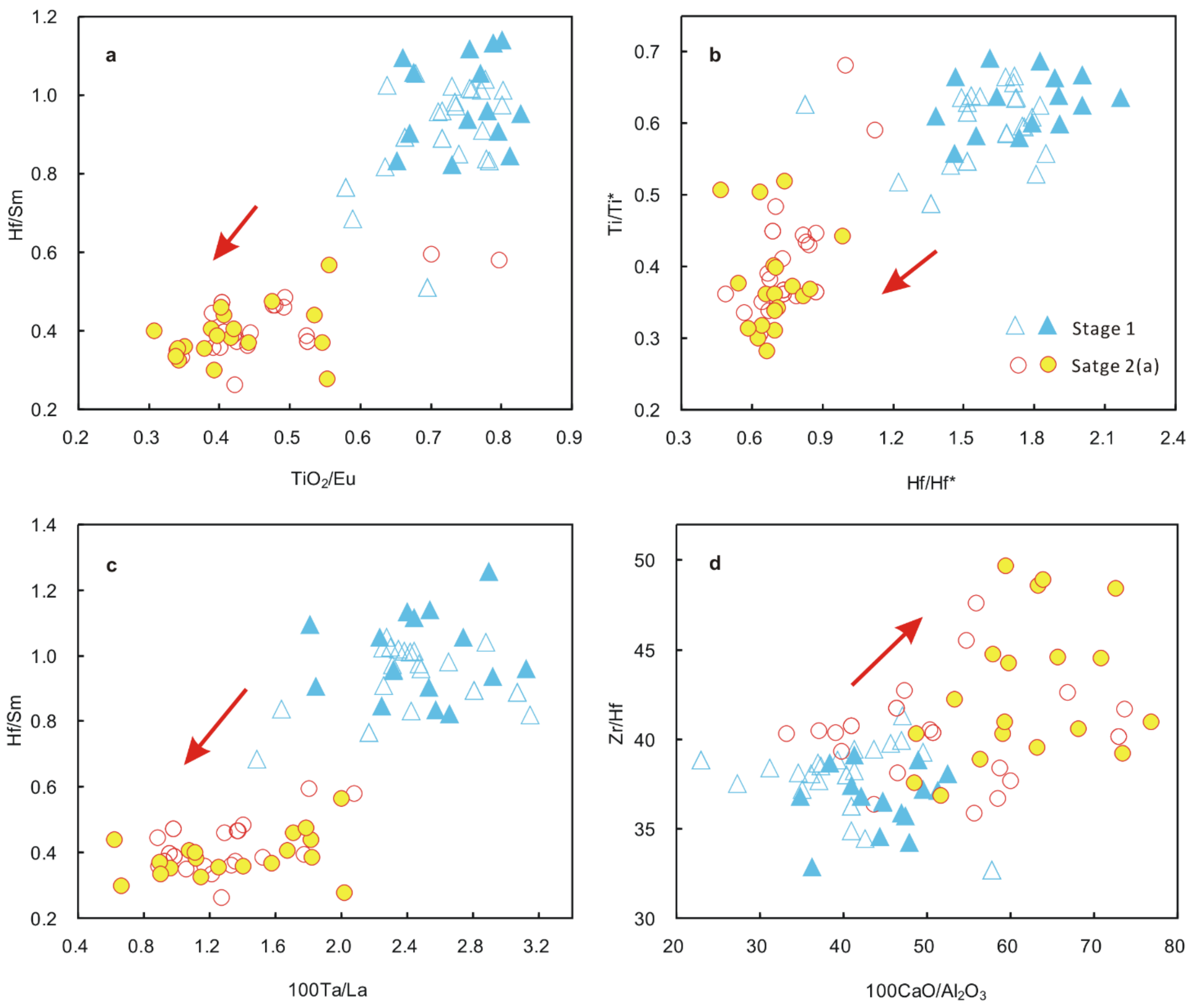

Supplementary Figure 2: Comparsions of major and trace element ratios between Stage 1 and Stage 2 (a), indicating that Stage 1 magmatic rocks result from metasomatism by the silicate-rich component whereas Stage 2 (a) are the results of carbonate-rich metasomatism. **a** Hf/Sm vs TiO_2/Eu; **b** Ti/Ti* vs Hf/Hf*; **c** Hf/Sm vs 100Ta/La; **d** Zr/Hf vs 100CaO/Al_2O_3. The meaning of the red arrows in **a** to **d** is as in Supplementary Figure 1, indicating that Stage 1 has lower Zr/Hf, Ca/Al, and higher Hf/Sm, Ti/Eu, Ti/Ti*, Hf/Hf*, Ta/La ratios than Stage 2 (a). Data sources and symbols are as in Supplementary Figure 1. The spatio-temporal distributions of Stage 1 and 2 (a) rocks are as in Fig. 1.

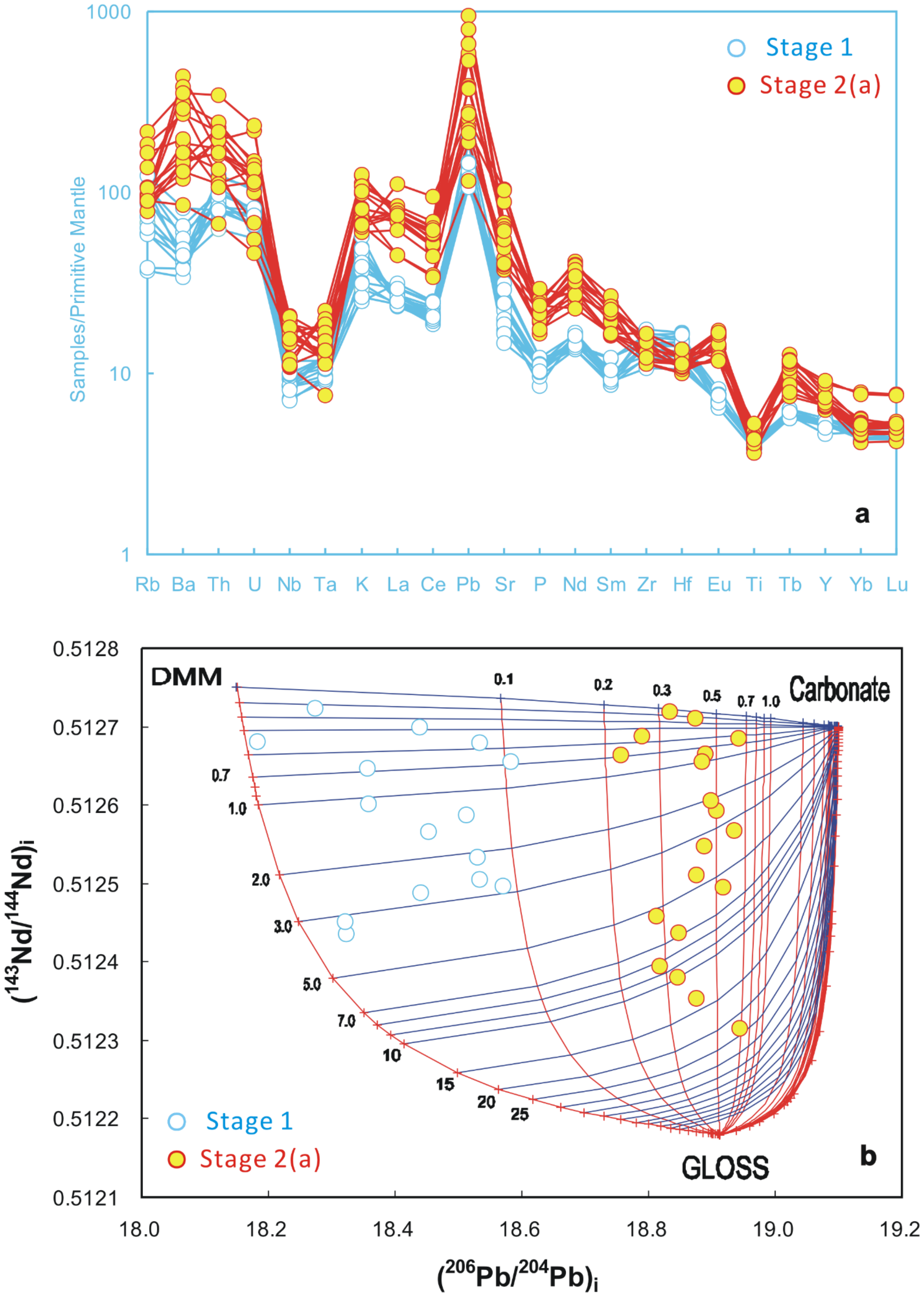

Supplementary Figure 3: Comparsions of mantle-normalized incompatible trace element patterns and Nd-Pb isotope compositions between Stage 1 and Stage 2(a), denoting the first-step transformation of magmatism in Tibet at 55 Ma. a Primitive mantle-normalized incompatible trace element diagrams; normalization factors are from ref.[11]. Stage 2 (a) show higher concentrations of incompatible trace elements and significant negative Zr-Hf-Ti anomalies whereas Stage 1 show lower concentrations of incompatible trace elements and positive Zr-Hf anomalies, and intermediate Ti anomalies. This further indicates that Stage 2 (a) result from mantle metasomatism by the carbonate component, whereas Stage 1 result from metasomatism by the silicate-rich component. All data plotted are from this study (see details in Supplementary Data 2). **b** $(^{143}Nd/^{144}Nd)_i$ vs $(^{206}Pb/^{204}Pb)_i$. The Pb-Nd isotope compositions of the Stage 1 and 2 (a) magmas plot among three end-members defined by depleted mid-oceanic ridge basalt (MORB) mantle (DMM), Global

Subducting Sediment (GLOSS) and India-derived carbonates, indicating a three-component mixing mantle source region. The mantle source of Stage 1 is mainly enriched by GLOSS because they plot almost along a linear array between the end-members DMM and GLOSS, whereas that of Stage 2 (a) is metasomatised by India-derived carbonates and GLOSS because they plot between them. This indicates a high proportation of India-derived carbonates in the mantle source of Stage 2 (a) but a low proportation of India-derived carbonates in the mantle source of Stage 1. This denotes that Stage 1 is a result of Neo-Tethys Oceanic lithospheric subduction whereas Stage 2 (a) result from an interaction (labelled mixed melts in Fig. 2c) between the southward spreading in the head of upwelling of CMP and GLOSS-rich mantle domains metasomatised by the NeoTethys-derived components in the mantle wedge. We thus propose that Stage 2 (a) result from a plume-wedge interaction. Data sources of end-members are as follows. DMM (depleted MORB mantle) is taken from references[11-12]. Because the Stage 2 magmatic rocks (Fig. 2) have been interpreted to result from upwelling of a mantle Transition Zone (MTZ)-derived carbonated asthenospheric mantle plume (CMP), therefore, in our modelling we have used OIB (ocean island basalts[11]) source mantle instead as the source material; this is assumed to have 0.05 times OIB[11] concentrations of the relevant trace elements (i.e. Sr=33.0 ppm, Nd=1.925 ppm and Pb=0.16 ppm) based on the assumption that OIB magmas are results of ~5% partial melting of their mantle source[13]. GLOSS (Global Subducting Sediment) is taken from ref.[14]. India-derived carbonates are taken from Supplementary Data 2. All data plotted are from this study (see details in Supplementary Data 2). The spatio-temporal distributions of Stage 1 and 2 (a) rocks are as in Fig. 1.

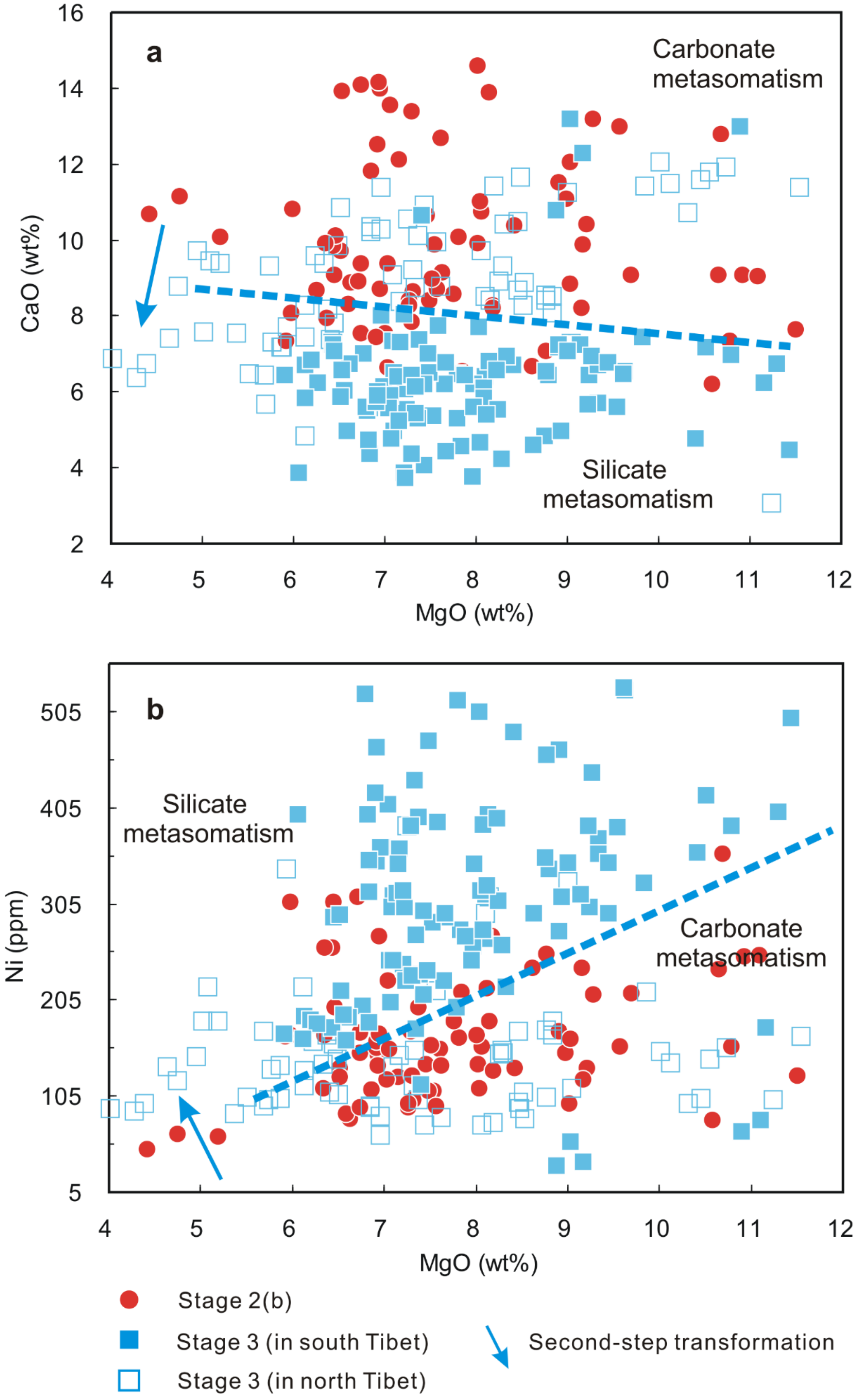

Supplementary Figure 4: Comparsions of selected major and trace element contents vs MgO (wt %) between Stage 2 (b) and Stage 3, indicating occurrence of the second-step transformation of magmatism in Tibet at 25Ma. a CaO (wt %) vs MgO (wt %). **b** Ni (ppm) vs MgO (wt %). Blue dashed lines in **a** and **b** separate silicate metasomatism from carbonate metasomatism[15]. Blue arrows in **a** and **b** denote the second-step transformation of magmatism at 25Ma, indicating decreases of the metasomatic carbonate component from Stage 2 (b) to Stage 3. Stage 2 (b) result from carbonate metasomatism whereas Stage 3 in south Tibet result from silicate metasomatism; Stage 3 in north Tibet result from carbonate and silicate-rich metasomatism. All data plotted are from Supplementary Data 2. The spatio-temporal distributions of Stage 2 (b) and Stage 3 rocks are as in Fig. 1.

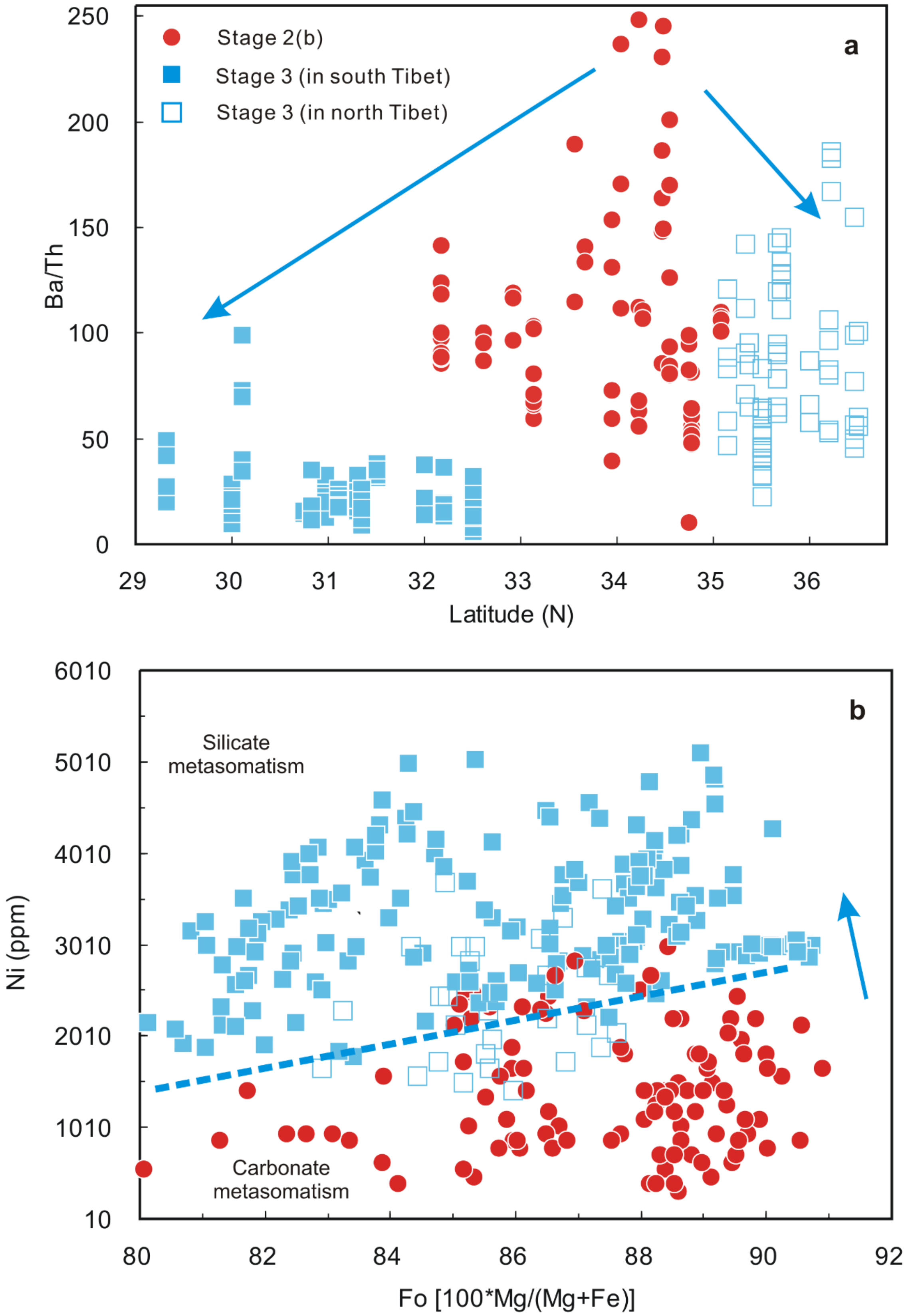

Supplementary Figure 5: Geochemical and mineralogic characteristics of Stage 2 (b) and Stage 3 magmas. a Ba/Th vs latitude, indicating decreasing trends of Ba/Th from central (Stage 2b) to north (Stage 3) and to south (Stage 3) Tibet, as shown by blue arrows. This denotes Stage 2 (b) result from carbonate metasomatism whereas Stage 3 in south Tibet result from silicate metasomatism; Stage 3 in north Tibet result from carbonate and silicate metasomatism. **b** Ni (ppm) vs Fo (mole %) of olivine phenocrysts, indicating that Stage 2 (b) result from carbonate metasomatism whereas Stage 3 in south Tibet result from silicate melt metasomatism; Stage 3 in north Tibet result from carbonate and silicate metasomatism. A blue dashed line in **b** separates silicate from carbonate metasomatism[16]. A blue arrow in **b** denotes the second-step transformation of magmatism at 25 Ma, indicating decreases of the metasomatic carbonate component from Stage 2 (b) to Stage 3. All data plotted in **a** are from Supplementary Data 1 and 2, whereas data in **b** are taken from Guo and Wilson (2019) [17] and references therein. The spatio-temporal distributions of Stage 2 (b) and Stage 3 rocks are as in Fig. 1.

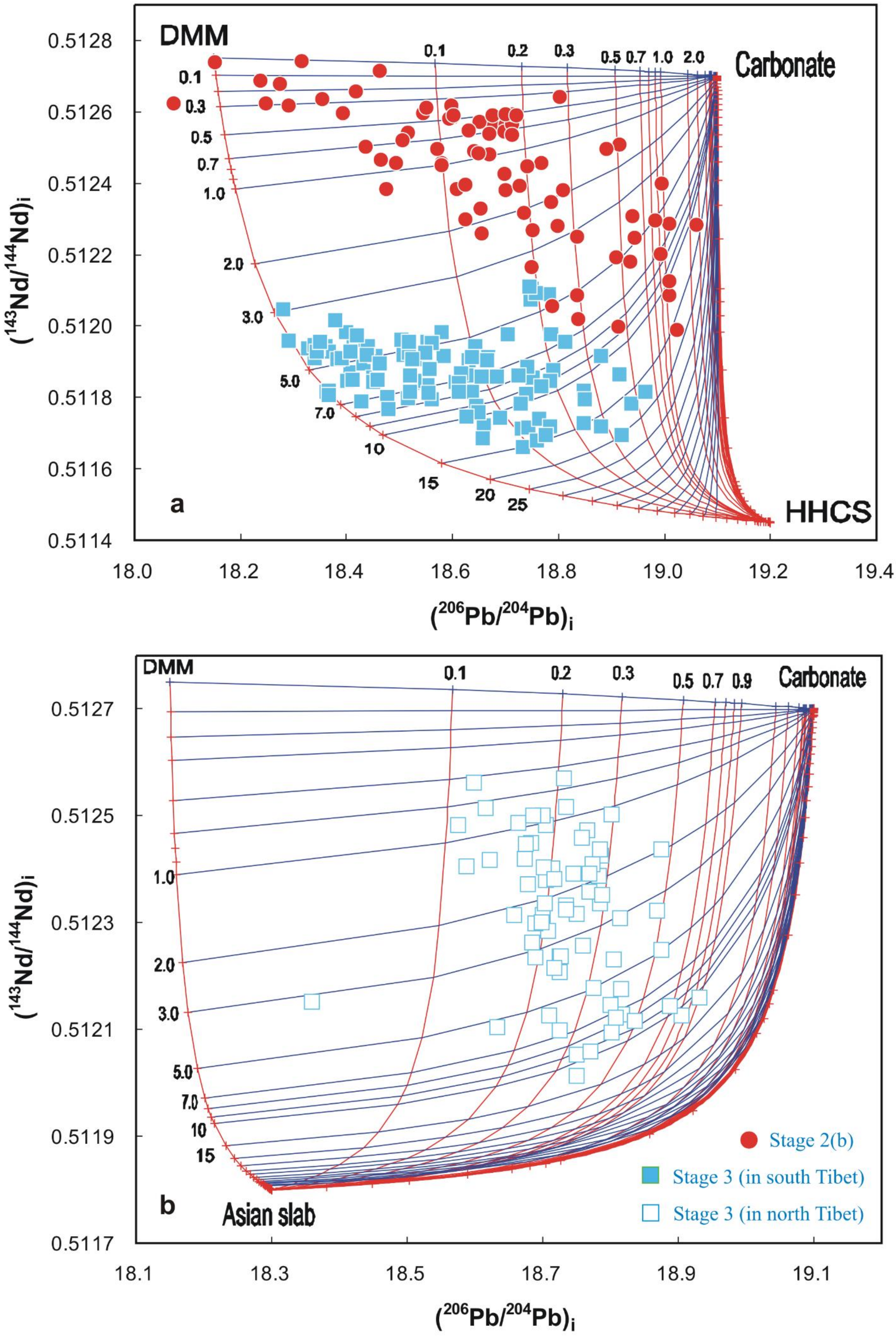

Supplementary Figure 6. Comparsions of Nd-Pb isotope compositions between Stages 2 (b) and Stage 3 magmas, indicating occurrence of the second-step transformation of magmatism in Tibet at 25Ma. a $(^{143}Nd/^{144}Nd)_i$ vs $(^{206}Pb/^{204}Pb)_i$ indicating a three-component mixing model between DMM, India-derived carbonates and India-derived Higher Himalayan Crystalline Sequence (HHCS) in the mantle sources of Stages 2 (b) and Stage 3. This indicates a high proportation of India-derived carbonates in the mantle source of Stage 2 (b) but a low proportion of India-derived carbonates in the mantle source of Stage 3. This indicates that the magmas of Stage 2 (b) and Stage 3 in south Tibet result from northward subduction of the Indian continental lithosphere. See Methods for detailed

modelling approach. **b** ($^{143}Nd/^{144}Nd)_i$ vs ($^{206}Pb/^{204}Pb)_i$ denoting that compositions of Stage 3 in north Tibet plot among three end-members defined by DMM, Asia-derived silicate sediments and India-derived carbonate sediments. This suggests a dual convergent subduction system of the India and Asia continents[17], leading to carbonate- and silicate-rich metasomatism in the mantle source region of Stage 3 in north Tibet. All data are taken from Supplementary Data 2. The spatio-temporal distributions of Stage 2 (b) and Stage 3 rocks are as in Fig. 1. Data sources of end-members: DMM is taken from references[11-12]; India-derived carbonates, India-derived HHCS and Asia-derived silicates are taken from Table 2.

Supplementary References

[1] Le Maitre, R. W., Bateman, P., Dudek, A., Keller, J., Lameyre, J., Le Bas, M. J., Sabine, P. A., Schmid, R., Sorensen, H., Streckeisen, A., Woolley, A. R. & Zanettin, B. *A classification of igneous rocks and a glossary of terms*. Blackwell Scientific, Oxford, 1-236 (1989).

[2] Rickwood, P. C. Boundary lines within petrologic diagrams which use oxides of major and minor elements. *Lithos* **22**, 247–263 (1989).

[3] Mo, X. et al. Mantle contributions to crustal thickening during continental collision: Evidence from Cenozoic igneous rocks in southern Tibet. *Lithos* **96**, 225-242 (2007).

[4] Mo, X. et al. Contribution of syncollisional felsic magmatism to continental crust growth: A case study of the Paleogene Linzizong volcanic Succession in southern Tibet. *Chem. Geol*. **250**, 49-67 (2008).

[5] Lee, H. Y. et al. Geochemical and Sr–Nd isotopic constraints on the genesis of the Cenozoic Linzizong volcanic successions, southern Tibet. *J. Asian Earth Sci*. **53**, 96-114 (2012).

[6] Yan, H. et al. Arc andesitic rocks derived from partial melts of mélange diapir in subduction zones: evidence from whole-rock geochemistry and Sr-Nd-Mo isotopes of the Paleogene Linzizong volcanic succession in southern Tibet. *J. Geophys. Res. Solid Earth* **124**, 456-475 (2019).

[7] Zhang, S. Q. *Cenozoic Linzizong volcanic rocks in south Tibet: implications for petrogenesis and deep process*. Ph. D. dissertation. Beijing: China University of Geosciences 1-120 (1996).

[8] Dong, G. C. *Linzizong volcanic rocks in Linzhou volcanic basin, Tibet: implications for India-Eurasia collision process*. Ph. D. dissertation. Beijing: China University of Geosciences (2002).

[9] Yue, Y. H. & Ding, L. $^{40}Ar/^{39}Ar$ Geochronology, geochemical characteristics and genesis of the Linzhou basic dikes, Tibet. *Acta Petrol. Sin*. **22**, 855-866 (2006).

[10] Liu, A. L. et al. Origin of the ca. 50 Ma Linzizong shoshonitic volcanic rocks in the eastern Gangdese arc, southern Tibet. *Lithos* **304–307**, 374-387 (2018).

[11] Sun, S. & McDonough, W. F. Chemical and isotopic systematics of oceanic basalts: implications for mantle composition and processes. In: Saunders AD, Norry MJ (eds) *Magmatism in the Ocean Basins*. Geological Society, London, Special Publications **42**, 313–345 (1989).

[12] Workman, R. K. & Hart, S. R. Major and trace element composition of the depleted MORB mantle (DMM). *Earth Planet. Sci. Lett.* **231**, 53–72 (2005).

[13] Wilson, M. *Igneous Petrogenesis: A Global Tectonic Approach*. London: Unwin Hyman, 1-466 (1989).

[14] Plank, T. & Langmuir, C. H. The chemical compositions of subducting sediments and its consequences for the crust and mantle. *Chem. Geol.* **145**, 325-394 (1998).

[15] Guo, Z. F., Wilson, M., Zhang, M. L., Cheng, Z. H. & Zhang, L. H. Post-collisional ultrapotassic mafic magmatism in south Tibet: products of partial melting of pyroxenite in the mantle wedge induced by roll-back and delamination of the subducted Indian continental lithosphere slab. *J. Petrol.* **56**, 1365–1406 (2015).

[16] Ammannati, E., Jacob, D. E., Avanzinelli, R., Foley, S. F. & Conticelli, S. Low Ni olivine in silica-undersaturated ultrapotassic igneous rocks as evidence for carbonate metasomatism in the mantle. *Earth Planet Sci Lett* **444**, 64–74 (2016).

[17] Guo, Z. F. & Wilson, M. Late Oligocene–early Miocene transformation of postcollisional magmatism in Tibet. *Geology* **47**, 776–780 (2019).
