# Supplementary material for: India-Asia collision as a driver of atmospheric CO2 in the Cenozoic
Source: Nat Commun. 2021 Jun 23;12:3891. doi: 10.1038/s41467-021-23772-y (PMC8222363; doi:10.1038/s41467-021-23772-y)
Supplement: Supplementary file 2 — Description of Additional Supplementary Files [file 41467_2021_23772_MOESM2_ESM.doc]

**Description of Additional Supplementary Files**

**File Name**: Supplementary Data 1
**Description:** Ages of the Cenozoic magmatic rocks in the Tibetan Plateau. We have checked the data quality and compared the ages from the different methods (e.g. ^40^Ar/^39^Ar, K-Ar, zircon U-Pb) in order to further determine their ages. We do not collect the geochronological data from K-Ar dating method in previous studies if the samples have shown the corresponding age from ^40^Ar/^39^Ar and zircon U-Pb dating methods. We do not compile some geochronological data if the analytical results do not show the error values in much earlier previous studies. TL: thermoluminescence.

**File Name:** Supplementary Data 2
**Description:** Calculated processes and results of CCFM based on the geochemical compositions of the Cenozoic magmatic rocks in the Tibetan Plateau. The average age (Ma) in each volcanic field is taken from Supplementary Data 1. The upwelling rate of CMP (i.e. 50cm/year) is taken from Soltanmohammadi et al. (2018). Contents of Sr (=1927 ppm) and Nd (=741 ppm) are taken from Walter et al. (2008) and that of Pb (=125 ppm) is calculated based on 2% melting (Wang et al., 2017) using the average value of concentrations of Pb (=2.50 ppm) shown in India-derived carbonate in Supplementary Data 2, which are used as compositions of an end-member in Sr-Nd-Pb isotope modelling calculations. 1Pg=10^9^Tons.
